# Genes Differentially Expressed in Conidia and Hyphae of *Aspergillus fumigatus* upon Exposure to Human Neutrophils

**DOI:** 10.1371/journal.pone.0002655

**Published:** 2008-07-09

**Authors:** Janyce A. Sugui, H. Stanley Kim, Kol A. Zarember, Yun C. Chang, John I. Gallin, Willian C. Nierman, Kyung J. Kwon-Chung

**Affiliations:** 1 Laboratory of Clinical Infectious Diseases, National Institute of Allergy and Infectious Diseases, National Institutes of Health, Bethesda, Maryland, United States of America; 2 Department of Medicine, College of Medicine, Korea University, Anam-Dong, Seongbuk-Gu, Seoul, Korea; 3 Laboratory of Host Defenses, National Institute of Allergy and Infectious Diseases, National Institutes of Health, Bethesda, Maryland, United States of America; 4 J. Craig Venter Institute, Rockville, Maryland, United States of America; David Geffen School of Medicine at University of California Los Angeles, United States of America

## Abstract

**Background:**

*Aspergillus fumigatus* is the most common etiologic agent of invasive aspergillosis in immunocompromised patients. Several studies have addressed the mechanism involved in host defense but only few have investigated the pathogen's response to attack by the host cells. To our knowledge, this is the first study that investigates the genes differentially expressed in conidia vs hyphae of *A. fumigatus* in response to neutrophils from healthy donors as well as from those with chronic granulomatous disease (CGD) which are defective in the production of reactive oxygen species.

**Methodology/Principal Findings:**

Transcriptional profiles of conidia and hyphae exposed to neutrophils, either from normal donors or from CGD patients, were obtained by using the genome-wide microarray. Upon exposure to either normal or CGD neutrophils, 244 genes were up-regulated in conidia but not in hyphae. Several of these genes are involved in the degradation of fatty acids, peroxisome function and the glyoxylate cycle which suggests that conidia exposed to neutrophils reprogram their metabolism to adjust to the host environment. In addition, the mRNA levels of four genes encoding proteins putatively involved in iron/copper assimilation were found to be higher in conidia and hyphae exposed to normal neutrophils compared to those exposed to CGD neutrophils. Deletants in several of the differentially expressed genes showed phenotypes related to the proposed functions, i.e. deletants of genes involved in fatty acid catabolism showed defective growth on fatty acids and the deletants of iron/copper assimilation showed higher sensitivity to the oxidative agent menadione. None of these deletants, however, showed reduced resistance to neutrophil attack.

**Conclusion:**

This work reveals the complex response of the fungus to leukocytes, one of the major host factors involved in antifungal defense, and identifies fungal genes that may be involved in establishing or prolonging infections in humans.

## Introduction

Invasive aspergillosis (IA) is one of the leading causes of mortality in immunocompromised patients, with *Aspergillus fumigatus* being the most frequent etiologic agent [1], [2]. IA usually develops after germination of the inhaled conidia, which then grow into mycelia in the lungs. In immunocompetent individuals, both phagocytes, alveolar macrophages and neutrophils, arrest conidial growth [3], [4] whereas defenses against hyphae depend on neutrophils. Neutrophils are recruited to the site of infection to phagocytize and kill pathogens. Phagocytosis can trigger the production of cytotoxic reactive oxygen species (ROS) by the enzymatic complex NADPH oxidase and the fusion of cytoplasmic granules with vacuoles containing pathogens. Mutation in any of the four components of the NADPH oxidase complex, as is the case in patients with chronic granulomatous disease (CGD), leads to deficient production of superoxide ions, hydrogen peroxide, hydroxyl anions and hypohalous acid, resulting in increased predisposition to recurrent bacterial and fungal infections [5], [6], [7]. Both, clinical evidence and in vitro data, show that neutrophils from CGD patients, unlike neutrophils from healthy individuals, are not able to damage *A. fumigatus* hyphae, suggesting a dependency on ROS [8]. In contrast, CGD neutrophils are as efficient as normal neutrophils in inhibiting growth of *A. fumigatus* conidia indicating that the growth arrest occurs in an ROS-independent manner [4].

While much progress has been made in understanding how the host perceives fungal invasion and mobilizes the immune response, very little attention has been given to how *Aspergillus* alters itself in response to host attack. Such information could lead to the identification of metabolic pathways essential for pathogenesis and the design of novel targeted antifungal drugs to counter this pathogen. Although a few genes, such as *alb1* involved in melanin synthesis [9], [10], *gliP* involved in the biosynthetic pathway of gliotoxin [11], [12] and *laeA* a regulatory protein of secondary metabolites synthesis [13], have been shown to play a role in the virulence of *A. fumigatus*, little is known about the metabolic requirements or the compensatory defense mechanisms that enable survival of the fungus inside the host.

Using genome-wide microarray, we aimed to identify genes differentially expressed in *A. fumigatus* conidia and hyphae following exposure to human neutrophils from either healthy individuals (normal) or CGD patients. Our findings indicate that oxidative stress responses, reductive iron assimilation and metabolic reprogramming are all mechanisms employed by *A. fumigatus* to attempt survive within the host environment.

## Materials and Methods

### Strains

*A. fumigatus* clinical isolate B-5233 was maintained on *Aspergillus* minimal medium [10]. Conidia from 7 day-old cultures were harvested with 0.01% Tween-20 in PBS and washed twice with water. Unless specified, the conidial suspensions were prepared in RPMI/HEPES [RPMI 1640 buffered with 25 mM HEPES, pH 7.4] and incubations were at 37°C with 5% C0_2_.

### Neutrophils

All human blood samples used in this study were collected after obtaining informed written consent from normal subjects (Protocol 99-CC-0168 approved by National Institutes of Health Internal Review Board) and patients with CGD (Protocol 93-I-0119 approved by National Institutes of Health Internal Review Board). Patients with clinically apparent infectious diseases were excluded. Blood was anticoagulated using acid citrate dextrose and neutrophils were purified as described previously [4]. The neutrophil preparations were typically about 95% pure.

### Viability of conidia

**Figure 1:**
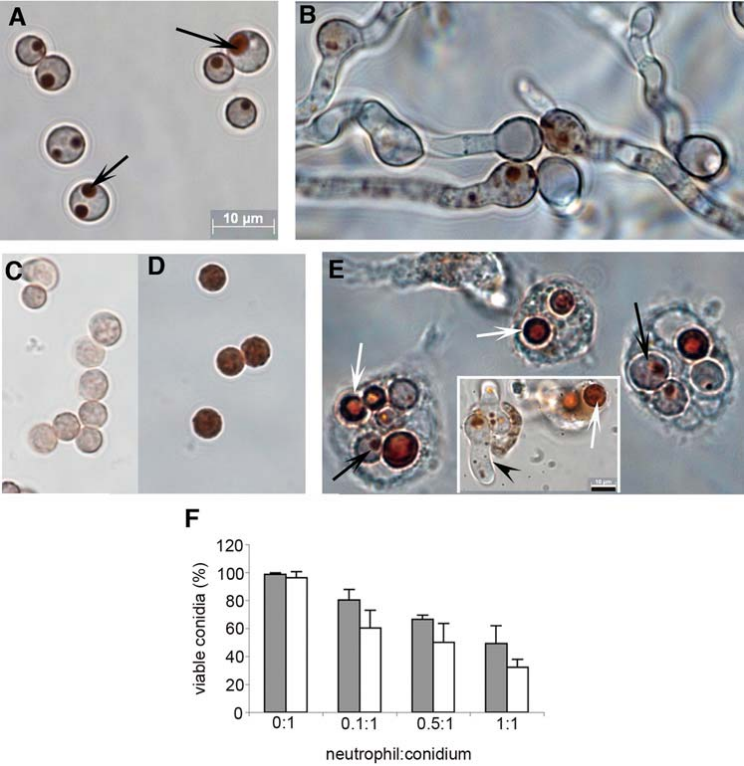
Effect of neutrophils on conidial viability. Conidial viability was monitored using the neutral red dye. (A–D) Control experiments to determine conidial viability based on patterns of staining with neutral red dye. (A) Conidia were incubated for 4 h at 37°C before the neutral red dye was added. Observation by bright field microscopy showed that conidia were able to take up and compartmentalize the dye (black arrows). (B) The same sample after 16 h showing extensive hyphal growth. (C and D) Conidia were incubated for 4 h at 37°C, heat-treated at 100°C for 20 min and stained with the dye. The conidia were not viable after the heat treatment. Some conidia stained faintly (C) while others stained deeply without compartmentalization of the dye (D). (E) Conidia exposed to neutrophils. Conidia were incubated for 4 h at 37°C, exposed to CGD neutrophils (0.5∶1 neutrophil to conidia) for 2 h and stained with neutral red. Non-viable conidia appeared as entirely red (white arrows) similar to the heat treated conidia observed in (D), whereas viable conidia showed the compartmentalization of the dye (black arrows) as seen in the control (A). The magnification of the figures (A–E) is the same. Magnification bar of the inset in E is 10 µm. The conidia shown in C and D are smaller than those shown in A due to shrinkage caused by heat-treatment. Further incubation for 2 h showed conidia that compartmentalized the dye were able to germinate and develop hyphal filament (inset in E, black arrowhead). (F) Conidia were challenged with normal (gray bars) or CGD (white bars) neutrophils at different ratios and the viability was monitored using neutral red after 2 h exposure. Data presented are mean% viability±SD from four experiments using different donors.

The neutral red dye was used to assess conidial viability upon exposure to neutrophils. A microscopic assay was developed to determine the viability of individual conidia internalized by host cells. Neutral red, a vital dye that requires cellular energy for active uptake was used as a viability indicator [14], [15], [16], [17], [18]. First, however, control experiments were performed to ascertain the viability of conidia that displayed different staining patterns of the neutral red dye. The first control was performed to determine whether conidia that were treated with high temperature were still viable. Conidia were incubated in RPMI/HEPES for 4 h before incubation at 100°C for 20 min. Heat-treated conidia failed to germinate or develop into hyphae upon further incubation in fresh RPMI/HEPES at 37°C, indicating that they were non-viable. The second control was performed to characterize the patterns of neutral red staining in conidia incubated at 100°C. Conidia were heat-treated as mentioned above and the neutral red dye (Matheson Coleman & Bell, USA) was added to a final concentration of 0.003%. The stained conidia were observed by bright-field microscopy. In the untreated control, conidia were able to compartmentalize the dye (Fig. 1A, black arrows). In the heat-treated conidia (Fig. 1C and D) two different patterns were observed: the conidia were either unstained showing clear cytoplasm or the dye was homogeneously distributed throughout the conidial cytoplasm without any evidence of compartmentalization. Based on these observations, the conidia were scored as viable only when they were able to compartmentalize the dye. The ratios of neutrophil to conidia tested were 0∶1, 0.1∶1, 0.5∶1 and 1∶1. 300 µl of conidial suspension (2 ×10^6^ conidia/ml) was added to the wells of 4-well chambered cover glass slides (Lab-Tek, USA) and incubated for 4 h prior to exposure to neutrophils. 175 µl of RPMI/HEPES containing the neutrophils required for each ratio was mixed with 25 µl of pooled human plasma from citrated human blood (Bioreclamation, USA), added to the chamber wells and incubated for additional 2 h. Because neutrophils can release DNA when activated [19], 100 µl of bovine DNase I (Sigma) [10 U/ml in 10 mM Tris pH 7.5, 2.5 mM MgCl_2_, 0.5 mM CaCl_2_] was added to the chamber wells to prevent any unknown interaction of dye and DNA. After 20 min incubation with DNase I at room temperature, neutral red (0.003%) was added and the samples observed by bright-field microscopy to score the viability of conidia within neutrophils.

### Challenge of *A. fumigatus* with neutrophils

**Experiments with conidia**: To ensure that the RNA was isolated prior to emergence of the germ tube, conidial incubation was limited to 4 h. 1×10^8^ conidia suspended in 10 ml of RPMI/HEPES were inoculated into Petri plates and allowed to swell for 2.5 h prior to addition of neutrophils. 4.25 ml of RPMI/HEPES containing 2.5×10^7^ neutrophils was mixed with 0.75 ml of pooled human plasma and added to the plates. The plates were then incubated for an additional 1.5 h. Conidia and neutrophils were harvested in 1% Igepal-CA-630 (Sigma) to lyse the neutrophils and centrifuged at 12000 *g*, at 4°C for 5 min. Conidia were collected, frozen and lyophilized. Three biological replicates with neutrophils from normal as well as CGD neutrophils were carried out. In each biological replicate, the neutrophils were from a single donor. The biological replicates with normal and CGD neutrophils are referred as N1-N3 and C1-C3, respectively. **Experiments with hyphae**: 5×10^6^ conidia suspended in 10 ml of RPMI/HEPES were inoculated into Petri plates and incubated for 8 h prior to addition of neutrophils. After 8 h of incubation, a majority of the conidia had germinated and formed hyphal filaments. 4.25 ml of RPMI/HEPES containing 5×10^6^ neutrophils (a ratio of 1∶1 of neutrophil to conidia) was mixed with 0.75 ml of pooled human plasma and added to the plates, which were then incubated for additional 75 min. Previously we have shown that 2 h of interaction between *A. fumigatus* hyphae (strain B-5233) and neutrophils was sufficient to cause significant hyphal damage [4] which was not apparent at 75 min interaction (data not shown). Therefore, to avoid extensive hyphal damage and yet allow enough time for the fungal cells to respond to neutrophils, the interaction was fixed at 75 min. Hyphal cells were harvested as described above. Four biological replicates with normal neutrophils and three biological replicates with CGD neutrophils were carried out. In each biological replicate, the neutrophils were from a single donor. The biological replicates with normal and CGD neutrophils are referred as N4-N7 and C4-C6, respectively. As a reference control, conidia or hyphae were incubated with plasma without neutrophils. RNA was isolated from the fungal cells using Trizol reagent (Invitrogen, USA) and purified with the RNeasy kit (Qiagen, USA). The RNA samples were used for microarray analysis and quantitative real time-PCR (qRT-PCR) assays. For the qRT-PCR assays, the RNA was treated with Turbo DNase (Ambion, USA) according to the manufacturer's protocol.

### Transcriptome profiling with the whole genome microarray

To identify the *A. fumigatus* genes that are differentially expressed in response to human neutrophils, we used *A. fumigatus* strain Af293 DNA amplicon microarrays containing 9,516 genes [20]. Hyphae and conidia, representing the two developmental stages of the fungus, were examined separately in this study. RNA from hyphae or conidia incubated in plasma in RPMI/HEPES without neutrophils was used as reference, and was co-hybridized with the query samples obtained from the corresponding sets incubated with neutrophils. Each biological replicate was carried out with neutrophils obtained from a different donor, and the RNA samples associated with each neutrophil source was paired separately with a corresponding reference sample prepared at the same time. RNA samples were labeled with Cy3 or Cy5 fluorescent dye and the collection of 13 hybridizations (Table 1) was replicated with dye labels reversed, except for samples N6 and C4. The labeling reactions with RNA and the hybridizations were performed according to protocols described in the J. Craig Venter Institute (JCVI) standard operating procedures <http://pfgrc.jcvi.org/index.php/microarray/protocols.html>. Hybridized slides were scanned using the Axon GenePix 4000B microarray scanner and the TIFF images generated were analyzed using the JCVI Spotfinder program (<http://www.pfgrc.tigr.org/tools.shtml>, JCVI, Rockville, MD) to obtain relative levels of transcript. Data was normalized using a local regression technique LOWESS (LOcally WEighted Scatterplot Smoothing) for hybridizations using the software MIDAS (<http://www.pfgrc.tigr.org/tools.shtml>, JCVI, Rockville, MD). The resulting data was averaged from triplicate copies of the genes printed on each array and from duplicate flip-dye arrays for each experiment, taking a total of 6 intensity data points for each gene (Accession number pending). Genes were grouped based on their expression vectors using the k-means algorithm, and selected groups of interest were again organized for similar expression patterns using Euclidean distance and hierarchical clustering with the average linkage clustering method JCVI MeV (<http://www.pfgrc.tigr.org/tools.shtml>, JCVI, Rockville, MD). The Significance Analysis of Microarrays (SAM) [21] method was used to identify genes differentially expressed between fungal cells exposed to normal vs CGD neutrophils.

**Table 1:** Design for the microarray hybridizations

		Cy5			
		hyphae in plasma with normal neutrophils	hyphae in plasma with CGD neutrophils	conidia in plasma with normal neutrophils	conidia in plasma with CGD neutrophils
**Cy3**	hyphae in plasma	3*	3*		
	conidia in plasma			4*	3*

* represents the number of donors (biological replicates). Each biological replicate was carried out with neutrophils from a single donor.

### Gene expression under glucose-limited conditions

RNA was isolated from swollen conidia that were incubated in media G^−^ alone [RPMI 1640 without glucose, buffered with 25 mM HEPES, pH 7.4] or G^−^ supplemented with either 10 mg/ml glucose (G+) or 10 µl/ml of a fatty acid mixture (FA) containing linoleic, oleic, myristic, lauric and arachidonic acids (Sigma). 10^8^ conidia were suspended in 10 ml of RPMI/HEPES, inoculated into a filtration unit [50 mm diameter filter with pore size 0.2 µm (Nalgene, USA)] and incubated for 2.5 h to allow conidial swelling. Since the filtration unit was not connected to a vacuum system, filtration did not occur during the 2.5 h incubation. The unit was then connected to a vacuum system to filter the media. The membrane was washed with 100 ml of water and the unit disconnected from the vacuum system. Ten milliliters of G^−^, G+ or FA was added to the filter and incubated for additional 1.5 h before conidia were harvested to isolate the RNA described as above.

### Quantitative Real Time-PCR (qRT-PCR)

The RNA was reverse transcribed using the High Capacity cDNA Archive kit (Applied Biosystems, USA) according to manufacturer's instructions. 5 µl of the first-strand cDNA reaction (corresponding to 25 ng of initial RNA used for cDNA synthesis) was added to 20 µl of the qRT-PCR mix [12.5 µl of TaqMan® Universal PCR Master Mix (Roche Molecular Systems, CA, USA), 2.25 µl of 10 µM Forward and Reverse primers each, 0.0625 µl of 10 µM probe and 2.93 µl of water]. The reaction was performed in an ABI Prism® 7700 Sequence Detection System. Total RNA was used as negative control. The genes glyceraldehyde 3-phosphate dehydrogenase (*gpdA*) and tubulin 2 (*tub2*) were amplified as endogenous control to standardize the amount of sample added to the reaction mix. Relative quantitation of gene expression was performed using the relative standard curve method. Results were expressed as relative fold-change. In the experiments with neutrophils the fold-change refers to the ratio between conidia exposed to neutrophils and non-exposed conidia, after normalization with *gpdA*. In the experiments with glucose-limited conditions, the fold-change refers to the ratio between conidia incubated in media G+ or FA and conidia incubated in media G−, after normalization with *tub2*. The gene *tub2* was chosen as normalization factor because the expression of *gpdA* could be directly affected by the glucose concentration in the media tested. Comparisons among the tested strains showed that the target genes had similar basal levels in media G+. The sequences of the primers and probes used for the qRT-PCR are listed on Table 2.

**Table 2:** Oligonucleotides used for the qRT-PCR assays.

Locus ID/Gene or Function	Probe	Primer Forward	Primer Reverse
Afu5g01970/*gpdA*	CCCCCATGTTCGTCATGGGTGTC	TCTCCGCTCCTTCTGCTGAT	CGGAGGTGTAGGTGGTGTTGT
Afu7g00250/*tub2*	CGCCGCAAAGCCTTCTTGCACT	CCCAGTTCACGGCCATGT	CCATTCCCTCGCCGGTATA
Afu6g02800/GPI-anchored protein	TCGGCAGCCGCACCAGTGA	GTGGATTCGTTGGCATTGC	TGGCATTTCGCCTATTGCA
Afu6g02810/low affinity copper transporter	CGCCGTTACATCGCCATAGCGG	ACTGGTTTGCGACTCATCTCAA	CGACCGGCTTCAGATGATTT
Afu6g02820/metalloreductase, putative	CCCCCGATAACGATTCGCGGTC	GAAGCGAGCTATGCGACGTT	CAGAGGAGCAAAGTGGAAGGAT
Afu6g13750/ferric-chelate reductase, putative	CACGGTAAATGGCGCCTCCCC	GGAGTTGGAGCCCATGCA	ACTGAGTCTACTGCCTCCCAAGA
Afu2g03730/high affinity copper transporter family prot	CTCCACAACCGCCTCGTCCCAAG	CGCCCGCCTAAGAAATCAG	CATCGGTGGCACGGGTTA
Afu6g04920/NAD-dependent formate dehydrogenase (*fdh*)	TCCCAGCGACGCAGCCCG	TTCGTGCACCAGAGATGGAA	TCCCACCAGCAGTAGTCCATT
Afu4g13510/isocitrate lyase AcuD (*icl*)	TTGGCTCTACCAATCCCAACCTGCAG	CGCGATCACGCCTTCATC	GCCAGCATGAGGTCGTTCA
Afu6g07740/peroxisomal biogenesis factor (*pex11*)	AAGCTCGCTACCGCCAAGCGG	TGGACGTGATCGGCGTTAA	AACGGTAGGCAGACTCTTGCA
Afu3g02270/mycelial catalase (*cat1*)	CAGGACGCCGACCTTGAGGCC	GTTGCCGTACTTTTGCTTGATG	TGGAACCAAGCTGAAGAAGCTT
Afu8g01670/bifunctional catalase-peroxidase (*cat2*)	TCTGGACAAAGCTCGTCGTCTGCTCTG	AGCTGGCCCGACAATGTC	CCGGGTGCTGGACAGAAC
Afu8g04130/C6 transcription factor Ctf1B (*farB1*)	GCCCGCCGTCATCATCCACCTA	GGTCGCTCGATTTGATATCGA	ACCGGCGTCACTGTGCTT
Afu4g03900/multifunctional beta-oxidation protein (*mfp*)	TCTGGTCGATCCCGAACCTGTCG	GCATCCGTGGTGGTTAACG	TTTCCACCCATCTTCTTGATCTC
Afu4g03960/C6 transcription factor Ctf1A (*farA*)	CCTGATGGCACGGCCATATTCCC	ACTTCCAGCCAGACCAGTTGTT	TTGCGGAGACGCCAACTC

### Deletion of *farA* (Afu4g03960) and *farB* (Afu8g0413 and Afu1g00410)

**Deletion of** ***farA*** **:** a vector was constructed by inserting the hygromycin resistance cassette between a 1,010-bp fragment located upstream and a 496-bp fragment located downstream of the coding region of the *farA* gene. **Deletion of** ***farB***: because the flanking regions adjacent to the two *farB* genes, *farB1* and *farB2,* are identical, a deletion vector was built in such a manner that the two genes could be deleted by homologous integration of the vector at both loci simultaneously. The vector was constructed by insertion of the hygromycin resistance cassette between a 918-bp fragment located upstream and a 975-bp fragment located downstream of the coding region of the genes *farB*. The deletion constructs were then cloned into the pDHt/SK2 vector [22]. The deletion vector was integrated into the B-5233 genome via *Agrobacterium tumefaciens*-mediated transformation [22]. Southern hybridization identified two independent transformants (referred as JSA1 and JSA2) with homologous gene replacement resulting in deletion of *farA*. Likewise, two independent transformants (referred as JSB1 and JSB2) with homologous integration of the deletion vector in the *farB1* as well as *farB2* locus resulting in simultaneous deletion of both *farB* genes (data not shown) were identified.

### Growth assays

To evaluate whether deletion of *farA* or *farB* affected the growth of *A. fumigatus*, conidia were incubated on yeast nitrogen base, YNB (Difco, MD, USA) broth, supplemented either with 10 mg/ml glucose (YNB/G), 50 µl/ml FA (YNB/FA) or 0.26 M glycerol (YNB/GLY). 2×10^4^ conidia were inoculated into 200 µl of YNB/G, YNB/FA or YNB/GLY and the conidial suspension was added to the wells of an 8-wells chambered cover glass slide. The slide was incubated for 24 h before visualization under bright-field microscopy.

### Deletion of the genes encoding a copper transporter (Afu6g02810), a metalloreductase (Afu6g602820) and a putative GPI-anchored protein (Afu6g02800)

Since these three genes are located adjacent to each other on chromosome 6, a deletion vector was constructed to delete all three genes at once. The vector was constructed by insertion of the hygromycin resistance cassette between a 950-bp fragment and a 520-bp fragment flanking the coding regions of the first gene Afu6g02800 and the third gene Afu6g02820, respectively. The deletion constructs were then cloned into the pDHt/SK2 vector [22] and integrated into the B-5233 genome via *Agrobacterium tumefaciens*-mediated transformation [22]. Southern hybridization identified two independent transformants, T9 and T10, as the deletants of the three genes via homologous gene replacement (data not shown).

### Menadione treatment

To assay the effect of the oxidative agent menadione, 200 µl of 10^6^ conidia/ml was added to the wells of an 8-wells chambered cover glass slide and incubated for 8 h to allow germination and development of hyphal filaments. Menadione sodium bisulfite (Sigma), 40 µg/ml, was then added to the samples. The control wells received media instead of menadione. The slides were incubated for additional 24 h before visualization by bright-field microscopy (Zeiss Axiovert, Axiovison 4.0 software).

## Results

### Effect of neutrophils on conidial viability

We have shown previously that neutrophils from CGD and normal donors inhibit the growth of *A. fumigatus* conidia with equal efficacy [4]. Growth inhibition was assayed by monitoring the metabolic activity of mycelia that developed after the conidia were exposed to neutrophils. In this study, we used a dye, neutral red, to monitor changes in the viability of individual conidia exposed to neutrophils. Neutral red is a weak cationic dye that penetrates cells by nonionic diffusion and is compartmentalized by living cells in a process requiring cellular energy. This dye has been used as a cell viability indicator in mammalian cells [14], [15] and for the identification of lysosomes and vesicles in fungal species such as *Cryptococcus neoformans*, *Colletotrichum graminicola* and *Botrytis cinerea* [16], [17], [18]. In control experiments, we observed that conidia capable of compartmentalizing the dye were able to germinate and develop into hyphae (Fig.1A,B) whereas conidia that failed to compartmentalize the dye did not germinate (Fig. 1 C,D and data not shown). Figure 1E shows a micrograph of neutral red stained conidia exposed to CGD neutrophils. Assays with normal neutrophils showed similar results (data not shown). Germinating conidia, while phagocytized by neutrophils, showed compartmentalized neutral red whereas homogeneously stained conidia remained ungerminated (Fig. 1E inset). Based on these observations we scored the viability of conidia exposed to neutrophils and showed that conidial viability decreased in a neutrophil dose-dependent manner and that neutrophils from normal and CGD donors were equally capable of reducing conidial viability (Fig. 1F). This new method of scoring *Aspergillus* viability is consistent with previous findings based on metabolic activity of the fungus [4].

### Genes up-regulated in conidia upon exposure to neutrophils

**Figure 2:**
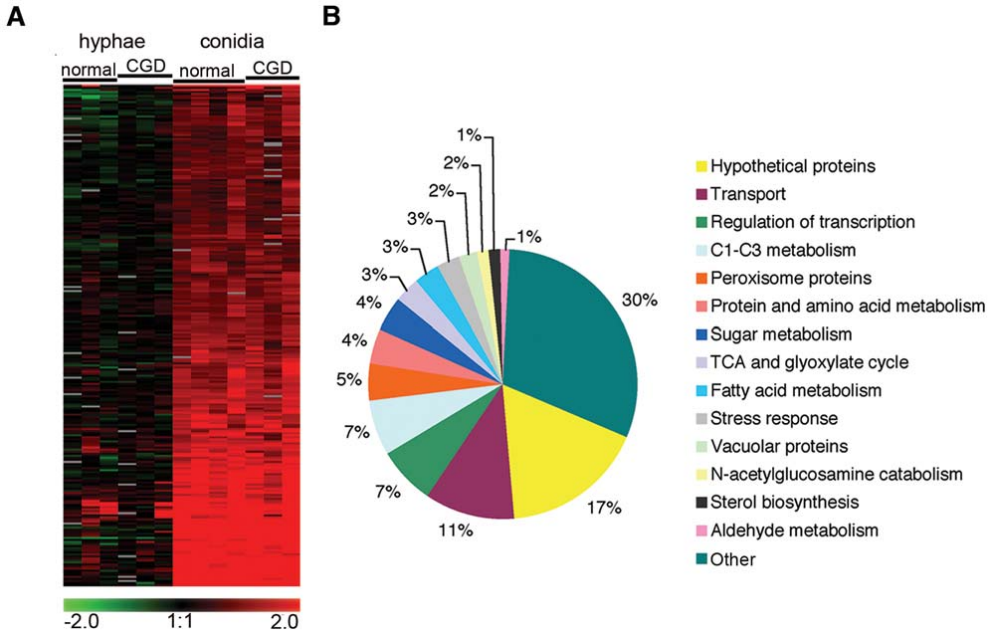
Differentially expressed genes in *A. fumigatus* exposed to neutrophils. (A) Gene clustering showing 244 genes differentially regulated between conidia and hyphae treated with normal or CGD neutrophils. Each column represents a biological replicate carried out with neutrophils from a single donor. (B) A general functional classification of the 244 genes is summarized in the pie chart.

**Figure 3:**
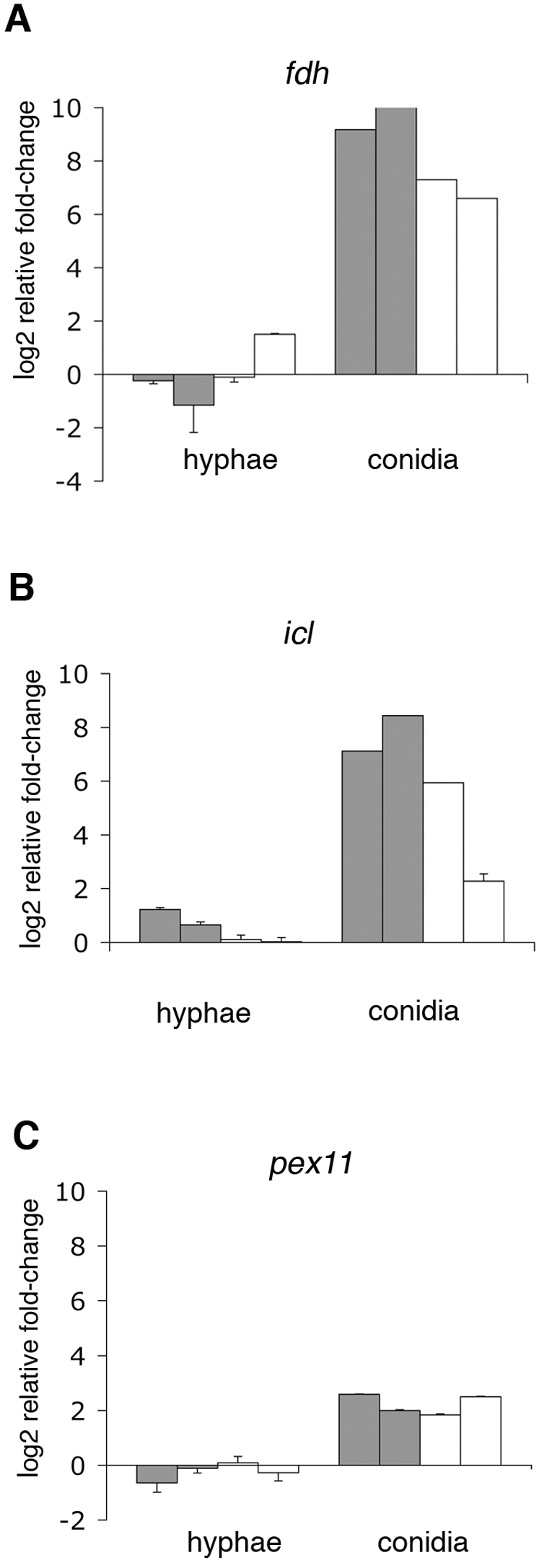
Expression level of genes differentially regulated between conidia and hyphae. The relative fold-change in the mRNA levels of the genes *fdh* encoding an NAD-dependent formate dehydrogenase (A), *icl* encoding an isocitrate lyase (B) and *pex11* encoding a peroxisomal biogenesis factor (C) were obtained by qRT-PCR. The assays were carried out with RNA isolated from hyphae and conidia exposed to neutrophils from two normal (grey bars) or two CGD (white bars) donors. Each bar represents a replicate carried out with neutrophils from a single donor (±SD of each qRT-PCR replicate). The relative fold-change represents the log2 ratio between fungal cells exposed to neutrophils and fungal cells without neutrophil challenge.

Since CGD neutrophils inhibited conidial growth as efficiently as normal neutrophils, we first focused on the identification of common genes that showed alterations of expression in the presence of either normal or CGD neutrophils. Conidia were exposed to neutrophils from normal or CGD donors and microarray analysis was carried out. To analyze the microarray data, genes were grouped based on their expression vectors using the k-means algorithm. The selected groups of genes were again organized for similar expression patterns using Euclidean distance and hierarchical clustering with the average linkage clustering method of JCVI MEV (see Materials and Methods). One of the most significant findings from the Clustering analysis was the identification of 244 genes up-regulated in conidia upon exposure to neutrophils (Fig. 2A, Table S1). Since most of these genes were up-regulated in conidia but not in hyphae, it is likely that these genes are part of a conidial-specific response. A general classification, based on the annotated functions, showed that the major groups consisted of genes involved in transport, regulation of transcription, metabolism of molecules with 1–3 carbons and peroxisomal proteins (Fig. 2B). Many of these genes are involved in the beta-oxidation of fatty acids, acetate metabolism, glyoxylate cycle and peroxisome biogenesis, suggesting a reprogramming of conidial metabolic pathways in response to neutrophil exposure (Table 3). In order to confirm changes in expression levels, we chose three of the up-regulated genes, *fdh*, *icl*, *pex11*, and analyzed their expression levels by qRT-PCR. The *fdh* gene, encoding an NAD-dependent formate dehydrogenase, is one of the genes that showed the highest increases in mRNA levels (average of 4-fold increase compared to control). The *icl* and *pex11* genes encode isocitrate lyase and the peroxisome biogenesis factor, respectively, which are putatively involved in fatty acid catabolism (Table 3). The qRT-PCR data confirmed that these genes were highly up-regulated only in conidia upon exposure to either normal or CGD neutrophils, (Fig. 3A–C).

**Table 3:** List of genes putatively involved in fatty acid beta-oxidation and acetate metabolism which were up-regulated in conidia upon exposure to neutrophils.

Locus ID	*Aspergillus fumigatus*	Homologous genes in *Aspergillus nidulans*
	Predicted function	(accession/gene)
	**Beta oxidation of fatty acids**:	
Afu8g04130	C6 transcription factor	AN1425.2/*farB1*
Afu1g00410	C6 transcription factor	AN1425.2/*farB2*
Afu1g14850	acyl-coA dehydrogenase	AN0824.2
Afu1g15170	acyl-coA thioesterase II	AN0868.2
Afu7g06100	acyl-coA dehydrogenase family	AN6752.2
Afu7g06090	fatty-acyl coA oxidase (Pox1)	AN6752.2
Afu4g10950	3-ketoacyl-coA thiolase peroxisomal A precursor	AN5646.2
Afu2g10920	enoyl-CoA hydratase/isomerase family protein	AN5916.2/*echA*
	**Acetate metabolism:**	
Afu1g13510	C6 transcription factor (FacB/Cat8)	AN0689.2/*facB*
Afu4g11080	acetyl-coenzyme A synthetase FacA	AN5626.2/*facA*
Afu6g14100	mitochondrial carnitine:acyl carnitine carrier	AN5356.2/*acuH*
Afu1g12340	carnitine acetyl transferase	AN1059.2/*facC*
Afu2g12530	carnitine acetyl transferase	AN6279.2/*acuJ*
	**Glyoxylate cycle:**	
Afu4g13510	isocitrate lyase AcuD	AN5634.2/*acuD*
Afu6g02860	isocitrate lyase	AN8755.2
	**Citric acid cycle:**	
Afu6g12930	aconitate hydratase, mitochondrial	AN5525.2
Afu3g07810	succinate dehydrogenase subunit Sdh1	AN2916.2
Afu4g12010	2-oxo acid dehydrogenases acyltransferase	AN3639.2
Afu5g10370	iron-sulfur protein subunit of succinate Sdh2	AN2332.2
Afu1g15590	succinate dehydrogenase subunit CybS	AN0896.2
Afu6g05210	malate dehydrogenase, NAD-dependent	AN6499.2
Afu4g00290	succinyl-CoA synthetase beta subunit	AN7000.2
	**Peroxisomal:**	
Afu6g07740	Peroxisome biogenesis factor (Pex11)	AN1921.2
Afu5g04310	Peroxisome membrane protein Pmp47	AN4280.2
Afu8g04780	Peroxisome membrane protein (PmpP24)	AN1483.2
Afu1g03560	Peroxisome biosynthesis protein (Peroxin-2)	AN4056.2
Afu1g04780	Peroxisome ABC transporter (Pxa1)	AN0426.2
Afu7g04260	Peroxisome biosynthesis protein (Peroxin-10),	AN5681.2
Afu2g10150	Peroxisome biosynthesis protein (Pas1/Peroxin-1)	AN5991.2

**Figure 4:**
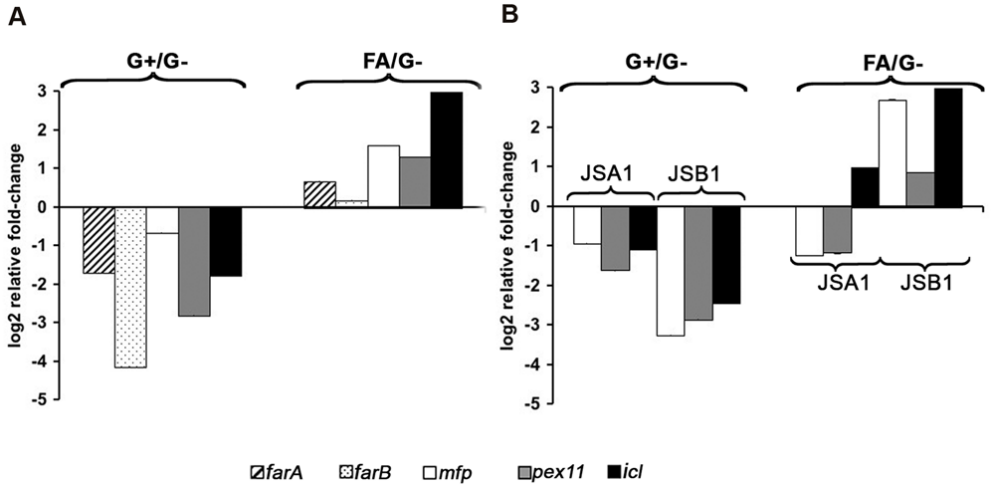
Expression level of the genes *farA*, *farB*, *mfp*, *pex11* and *icl* in the strains B-5233, JSA1 and JSB1. (A) Wild type strain B-5233. (B) Deletant strains of the *farA* and *farB* genes, JSA1 and JSB1, respectively. The relative fold-change represents the ratio between conidia incubated in media with glucose and media without glucose (G+/G-) or conidia incubated in media with fatty acid mixture, FA, and media without glucose (FA/G-). The genes *farA* and *farB* encode transcriptional regulators of fatty acid catabolism and *mfp* encodes a multifunctional enzyme involved in fatty acid catabolism. Error bars represents standard deviation of quantitative qRT-PCR. The experiment was repeated twice with similar results.

Hynes and collaborators have reported that fatty acid catabolism and peroxisomal function are both regulated by the genes *farA* and *farB* in *A. nidulans* [23]. Interestingly, our array data showed that two genes predicted to encode FarB (*farB1* and *farB2*) had their expression increased in conidia during the challenge with neutrophils (Table 3). Although the *farA* gene was not included in the array design, qRT-PCR assays with RNA isolated from neutrophil-exposed conidia showed that the mRNA levels of this gene increased in response to exposure to either normal or CGD neutrophils compared to mRNA levels from non-exposed conidia (data not shown). To assess whether *farA* and *farB* genes were involved in fatty acid catabolism in *A. fumigatus,* we analyzed the mRNA levels of these genes during glucose limitation, which has been reported to de-repress genes involved in fatty acid degradation [24], [25]. The genes *pex11*, *icl* and *mfp* were also included in this experiment. The *mfp* gene which encodes a multifunctional beta-oxidation enzyme involved in the degradation of fatty acids was also observed to be up-regulated in conidia exposed to either normal or CGD neutrophils (data not shown). To induce glucose-deprivation, germinating conidia of the wild type strain B-5233 were incubated in glucose-free medium, G^−^, and the RNA isolated. In parallel, germinating conidia were incubated in G^−^ media that was either supplemented with glucose (G+) or with a fatty acid mixture (FA). RNA was isolated and the expression of *farA*, *farB*, *pex11*, *icl* and *mfp* was assessed by qRT-PCR (Figure 4A). The changes in the expression were evaluated by comparing the mRNA levels in conidia incubated in G+ and in conidia incubated in G^−^. In figure 4A, the negative fold-changes in the ratios of G+/G^−^ indicated that the mRNA levels of the 5 genes were higher in the conidia incubated in G^−^ compared to conidia incubated in G+. In addition, the mRNA level of these genes was higher in conidia incubated in FA compared to conidia incubated in G^−^ (Fig. 4A, FA/G^−^). The results suggest that the expression of the genes *farA*, *farB*, *mfp*, *pex11* and *icl* is induced under glucose-limited conditions as well as in the presence of fatty acids.

To examine the potential role of fatty acid catabolism in the survival of *A. fumigatus* conidia, the genes *farA*, *farB1* and *farB2* were deleted. The deletants JSA1, JSA2, JSB1 and JSB2 were selected for the experiments. JSA1 and JSA2 are independent mutants generated by deletion of *farA*. JSB1 and JSB2 are independent mutants generated by double deletion of the genes *farB1* and *farB2*. qRT-PCR assays were performed to evaluate whether the deletion of *farA* and/or *farB* affected the transcriptional levels of *mfp*, *pex11* and *icl*. Germinating conidia from JSA1 and JSB1 were incubated in G^−^, G+ or FA before RNA was isolated. Similar to the observed with B-5233, conidia of both JSA1 and JSB1 exhibited higher mRNA levels of *mfp*, *pex11* and *icl* upon incubation in G^−^ compared to G+ (Fig. 4B, G+/G−). Incubation of conidia from JSA1 in FA, however, did not result in an increase of mRNA levels for these genes equivalent to that observed in B-5233, i. e., the levels of *mfp* and *pex11* were not significantly higher upon incubation in FA compared to G−. Although the mRNA level of *icl* showed some increase, it was still to a lesser extent than in B-5233. In the case of JSB1 conidia, the pattern of up-regulation was similar to that in B-5233 (Fig. 4B, FA/G−). Considering that FA is a mixture of fatty acids with chain-lengths from C12–C20, these results suggest that the gene *farA* plays a major role in the beta-oxidation of long-chain fatty acids. Similar patterns, supporting these findings, were observed with the mutant JSA2 (data not shown).

**Figure 5:**
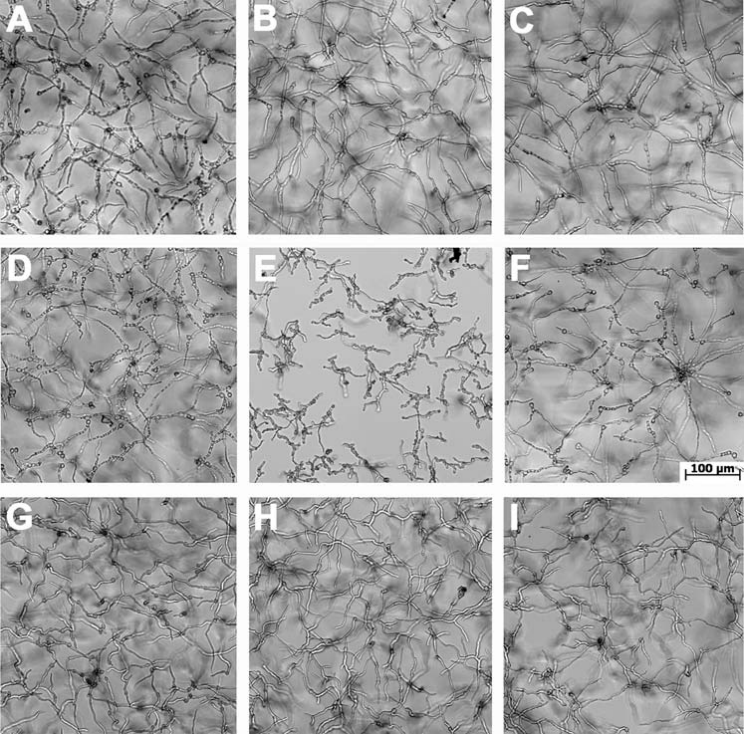
Growth comparison of the wild type strain B-5233 with the strains JSA1 and JSB1. JSA1 and JSB1 are mutant strains with deletion of the genes *farA* and *farB*, respectively. Conidia from B-5233 (A, D, G), JSA1 (B, E, H) and JSB1 (C, F, I) were inoculated in media supplemented with glucose (A–C), fatty acid mixture FA (D–F) or glycerol (G–I). After incubation for 24 h the samples were observed by bright-field microscopy. Magnification in F applies to all panels.

Since the qRT-PCR results suggested that *farA* was involved in the utilization of fatty acids during glucose-deprivation, we compared the growth of the mutant strains and the wild type in glucose, glycerol and FA. Figure 5 shows the growth of B-5233, JSA1 and JSB1. All 3 strains grew robustly on glucose (Fig. 5 A–C). The growth of mutant JSA1 in FA showed an extensive reduction whereas growth in glycerol was only slightly decreased (Fig. 5 E and H, respectively), supporting the hypothesis that *farA* is involved in the catabolism of long-chain fatty acids. In contrast, the growth of JSB1 was comparable to B-5233 either in FA or glycerol (Fig. 5 F and I, respectively). The mutant strains were then tested against normal neutrophils. The results showed that deletion of *farA* or *farB* did not increase susceptibility of conidia to neutrophils (data not shown). Our findings suggest that, although conidia appear to reprogram their metabolism towards fatty acid degradation, this metabolic route is not the only survival mechanism upon which *A. fumigatus* relies during neutrophil attack.

The clustering analysis also revealed a set of 558 down-regulated genes in conidia exposed to normal or CGD neutrophils. However, the average fold-change in the expression levels of a majority of these genes was smaller than a 2-fold decrease (data not shown). In addition, the genes in this cluster also showed a trend of down-regulation in hyphae which suggested that, unlike the 244 up-regulated genes, decreases in the mRNA levels of this cluster of genes was not specific for conidia.

### Genes differentially expressed in hyphae upon exposure to neutrophils

**Figure 6:**
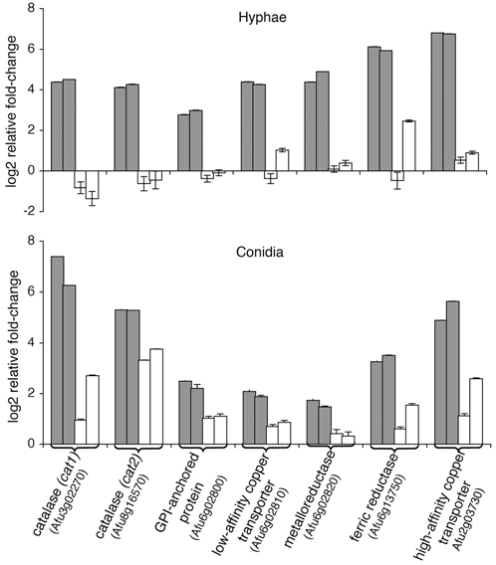
Expression levels of the genes encoding catalase Cat1, catalase Cat2, GPI-anchored protein, low-affinity copper transporter, metalloreductase, ferric reductase and high-affinity copper transporter in hyphae (A) and conidia (B) exposed to human neutrophils. qRT-PCR was performed with RNA from hyphae/conidia exposed to neutrophils from normal (grey bars) or CGD (white bars) donors. Each bar represents a replicate carried out with neutrophils from a single donor. Two normal and two CGD donors were used in each assay. The relative fold-change represents the log2 ratio between hyphae/conidia exposed to neutrophils and those without neutrophil challenge. Error bars represents standard deviation of qRT-PCR. The legend for the hyphae is the same as that for conidia.

*A. fumigatus* hyphae were exposed to either CGD or normal neutrophils and their transcriptome profiles were compared to identify differentially expressed genes. Considering that CGD neutrophils are deficient in ROS generation, we predicted that genes involved in oxidative response would show differential expression in hyphae exposed to normal versus CGD neutrophils. The profiles were compared using SAM with a median false discovery rate (FDR) of < 0.01%. Table 4 lists the genes differentially expressed in hyphae exposed to normal versus CGD neutrophils which showed an average relative fold-change ≥2. As expected, hyphae treated with normal neutrophils showed up-regulation of two genes encoding enzymes associated with oxidative stress responses, manganese superoxide dismutase (Afu1g14550) and catalase Cat1 (Afu3g02270). The *cat1* gene, together with *cat2*, has previously been described as mycelial catalase [26], [27]. Because the array data had missing values for *cat2* in three of the experiments, this gene was not included in the SAM results. However, a further examination of the array data prior to SAM analysis suggested that this gene showed a trend of up-regulation in hyphae exposed to normal neutrophils (Table 5). qRT-PCR assays confirmed that exposure to normal, but not CGD neutrophils caused an increase in the mRNA levels of *cat2* as well as *cat1* (Fig. 6A). Furthermore, a closer survey of the array data revealed that genes encoding glutathione peroxidase and thioredoxin reductase, both typically associated with oxidative stress, showed a trend of higher expression in hyphae exposed to normal neutrophils compared to CGD neutrophils (Table 5). The increase in the mRNA levels of genes associated with oxidative stress supports the notion that fungal hyphae are responding to ROS generated by normal neutrophils.

**Table 4:** Genes differentially expressed in hyphae exposed to normal vs CGD neutrophils^1^

Locus ID	Predicted function	log2 relative fold-change^2^
Afu1g14550	Manganese superoxide dismutase	4
Afu2g03730	High affinity copper transporter	6
Afu3g02260	Hypothetical protein	4
Afu3g02270	Catalase 1 (Cat1)	4
Afu5g02270	Fungal specific transcription factor domain family	2.5
Afu6g02800	GPI anchored protein, putative	11
Afu6g02810	Low-affinity copper transporter, putative	8
Afu6g02820	Metalloreductase	3
Afu6g13750	Ferric-chelate reductase	4
Afu6g13760	Alpha-1,2-mannosidase subfamily	4

1 : List contains the genes with statistically significant changes in expression (SAM) and fold-changes ≥2.

2 : Data are presented as the log2 of the average differences in the gene expression in hyphae exposed to normal neutrophils relative to CGD neutrophils.

**Table 5:** Relative expression of representative genes predicted to be involved in oxidative stress on fungal cells exposed to neutrophils.

Locus ID	Predicted function	log2 fold-change*												
		Hyphae						Conidia						
		N1	N2	N3	C1	C2	C3	N4	N5	N6	N7	C4	C5	C6
**Oxidative Stress**														
Afu3g12270	glutathione peroxidase	0.3	1.7	1.2	0.1	−0.1	0.7	1.5	1.9	2.1	1.9	0.7	0.4	0.5
Afu4g12990	thioredoxin reductase	0.6	1.2	1.9	0.1	0.0	##	1.0	1.3	1.2	1.2	−0.5	−0.3	##
Afu4g11580	Mn-superoxide dismutase	−0.1	0.1	0.0	−0.1	0.0	0.0	1.2	1.1	1.1	1.3	1.0	0.9	1.4
Afu1g14550	Mn-superoxide dismutase	1.5	2.6	2.2	nd	0.0	##	2.7	3.7	2.6	3.8	1.9	0.4	2.3
Afu3g02270	catalase (Cat1)	1.4	2.0	2.1	−0.4	nd	##	2.2	3.3	2.9	2.9	0.8	nd	0.2
Afu8g01670	catalase (Cat2)	0.3	2.0	2.1	nd	nd	nd	2.6	3.4	3.2	3.0	1.9	1.7	2.2

N1-7 and C1-6: Biological replicates performed with neutrophils from normal and CGD donors, respectively. In each biological replicate the cells were exposed to neutrophils from a single donor and the fungal RNA isolated.

nd: not detected

* ratio between fungal cells exposed to neutrophils and fungal cells not exposed to neutrophils.

**Figure 7:**
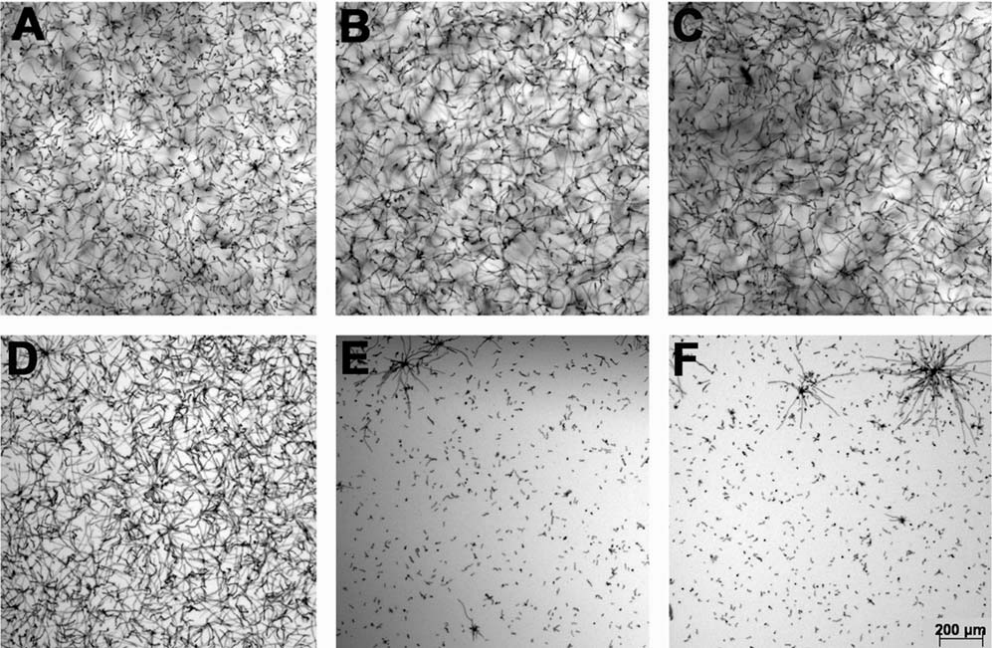
Effect of menadione on growth of the wild type strain B-5233 and the deletant strains T9 and T10. Conidia from B-5233 (A and D), T9 (B and E) and T10 (C and F) were pre-incubated for 8 h to allow germination before menadione was added. Samples were incubated for an additional 24 h and observed by bright-field microscopy. Panels A–C, no menadione added. Panels D–F, 40 µg/ml menadione. Magnification bar in F also applies to A–E.

Interestingly, four genes that are predicted to be involved in iron/copper assimilation were also differentially regulated. These genes encode two copper transporters (Afu2g03730 and Afu6g020810), one metalloreductase (Afu6g02820) and one ferric-chelate reductase (Afu6g13750) (Table 4). The genes encoding the copper transporter (Afug6g02810) and the metalloreductase (Afug602820) are located on chromosome 6 adjacent to the gene encoding a putative GPI-anchored protein (Afu6g02800) that was also differentially regulated (Table 4). Using qRT-PCR, we confirmed that the mRNA levels of these five genes were higher in hyphae exposed to normal neutrophils compared to CGD neutrophils (Fig. 6A). To evaluate whether some of these genes were relevant during interaction with neutrophils, we chose to delete the three genes that are located adjacent to each other on chromosome 6 (Afu6g02800-02820). Two independent strains, T9 and T10, containing a deletion of the three genes were selected for the experiments. Since one of the hypotheses proposes that these genes are involved in the resistance to oxidative stress, T9 and T10 were assayed with the oxidizing agents hydrogen peroxide and menadione. Menadione, a chemical that generates superoxide ion through enzymatic redox cycling, has been previously used to assess oxidative stress response in *A. fumigatus* and *Sacharomyces cerevisiae* [28], [29]. The mutant strains did not show an increased susceptibility to hydrogen peroxide (data not shown). However, in the presence of menadione, T9 and T10 were no longer able to germinate and develop mycelia while B-5233 grew robustly (Fig. 7). The defective growth of T9 and T10 suggests that one or more of the deleted genes are likely to be involved in resistance to menadione. However, in the interaction assays with normal neutrophils the mutant strains did not show an increased susceptibility to the phagocytes (data not shown). Our findings suggest that although these three genes are important for the response to the oxidative agent menadione, they are not essential for resistance to neutrophils. Although we were able to identify, by the SAM method, the genes whose expression levels were significantly higher in hyphae exposed to normal neutrophils relative to CGD neutrophils, we did not detect any genes whose expression levels were significantly lower (fold-change ≥2) in hyphae exposed to normal neutrophils.

### Genes differentially expressed in conidia upon exposure to normal vs CGD neutrophils

To identify genes differentially expressed in conidia we used the SAM method to analyze the transcription profiles of conidia exposed to normal versus CGD neutrophils. Using a FDR of < 0.01% and a relative fold-change ≥2, we identified a group of 8 genes that showed differential regulation (Table 6). Interestingly, the gene encoding the high-affinity copper transporter (Afu2g03730), which was identified as differentially regulated in hyphae, was also included in this group. To test whether the other 3 genes related to iron/copper assimilation and the gene encoding the GPI-anchored protein were also differentially regulated in conidia, qRT-PCR assays were performed. Although differences in the relative fold-change were smaller than those observed in hyphae, all five genes showed higher mRNA levels upon exposure to normal neutrophils compared to CGD neutrophils (Fig. 6B). In addition, genes encoding the glutathione peroxidase and the thioredoxin reductase, identified as differentially expressed in hyphae, were also found to be differentially regulated in conidia (Tables 5 and 6). A closer examination of the array data showed that the genes *cat1* and *cat2* and two manganese superoxide dismutase genes were also up-regulated in conidia exposed to normal as well as CGD neutrophils (Table 5). qRT-PCR assays showed that although the mRNA levels of *cat1* and *cat2* increased in response to both types of neutrophils, the levels were lower in conidia exposed to CGD neutrophils than to normal neutrophils (Fig. 6B).

**Table 6:** Genes differentially expressed in conidia in response to normal vs CGD neutrophils^1^.

Locus ID	Predicted function	log2 relative fold-change^2^
Afu2g03730	high affinity copper transporter	2
Afu3g02260	hypothetical protein	3
Afu4g10930	N-acetyltransferase superfamily	2.5
Afu3g12270	glutathione peroxidase family protein	2.5
Afu2g04060	NADH:flavin oxidoreductase	2
Afu4g12990	thioredoxin reductase	3
Afu5g02020	aldehyde reductase (GliO), putative	3
Afu5g09910	p-nitroreductase family	3

1 : List contains the genes with statistically significant changes in expression (SAM) and fold-changes ≥2.

2 : Data are presented as the log2 of the average differences in the gene expression in conidia exposed to normal neutrophils relative to CGD neutrophils.

## Discussion

The patterns of gene expression in fungal pathogens during interaction with host immune cells have been studied in several species such as *Candida albicans* and *Paracoccidioides braziliensis* [30], [31], [32]. Studies with *C. albicans* that were challenged with immune cells from human blood suggested that neutrophils induced gene expression more strongly than other cells [33]. In the present study, we have compared the transcriptome profiles of conidia and hyphae of *A. fumigatus* challenged with neutrophils from healthy donors and CGD patients. By exposing *A. fumigatus* to neutrophils from these two different types of donors, we were able to compare the response of the fungus between host cells that are capable of producing ROS with cells that are defective in ROS production. This system was suitable for identification of fungal genes that are up-regulated specifically in response to the ROS producing host cells.

The relatively recent appreciation of the important role of neutrophils in host defense against both conidia and hyphae of *A. fumigatus* prompted the present study. Bonnett and colleagues have shown that early recruitment and aggregation of oxidase-positive neutrophils, in addition to the phagocytic activity of alveolar macrophages, was essential in preventing conidial germination in the lungs of mice exposed to large numbers of *A. fumigatus* spores [3]. Furthermore, neutrophils from normal as well as CGD donors were shown to arrest conidial growth with equal efficiency and that lactoferrin, a major protein of neutrophil granules, contributes to reducing conidial growth [4]. Our findings suggest that upon internalizaton by neutrophils, conidia respond to changes in the environment by increasing the mRNA levels of genes putatively involved in the catabolism of fatty acids. The degradation of fatty acids is not a well described process in *A. fumigatus*. In *Aspergillus nidulans*, however, it has been described that the beta-oxidation of fatty acids occurs in peroxisomes as well as in mitochondria [34]. Two enzymes, the enoyl-coA hydratase and the multifunctional protein FoxA, which catalyze similar steps in the beta-oxidation of fatty acids are located in distinct organelles in *A. nidulans*. The enzyme enoyl-coA hydratase is localized in mitochondria whereas FoxA is in peroxisomes [34]. Considering that *A. fumigatus* conidia up-regulate genes encoding an enoyl-coA hydratase and a multifunctional protein Mfp, which are homologous to the genes encoding the *A. nidulans* enoyl-coA hydratase and FoxA, it is plausible that the degradation of fatty acid in conidia internalized by neutrophils occurs in the same way as in *A. nidulans*, i.e., the fatty acids are degraded in mitochondria as well as in peroxisomes. Other genes found to be up-regulated in *A. fumigatus* conidia upon exposure to neutrophils show homology to *A. nidulans* genes (Table 3) whose function has been characterized as being involved in acetate metabolism and the glyoxylate cycle. This observation reinforces the hypothesis that the genes up-regulated in *A. fumigatus* conidia are involved in fatty acid catabolism.

**Figure 8:**
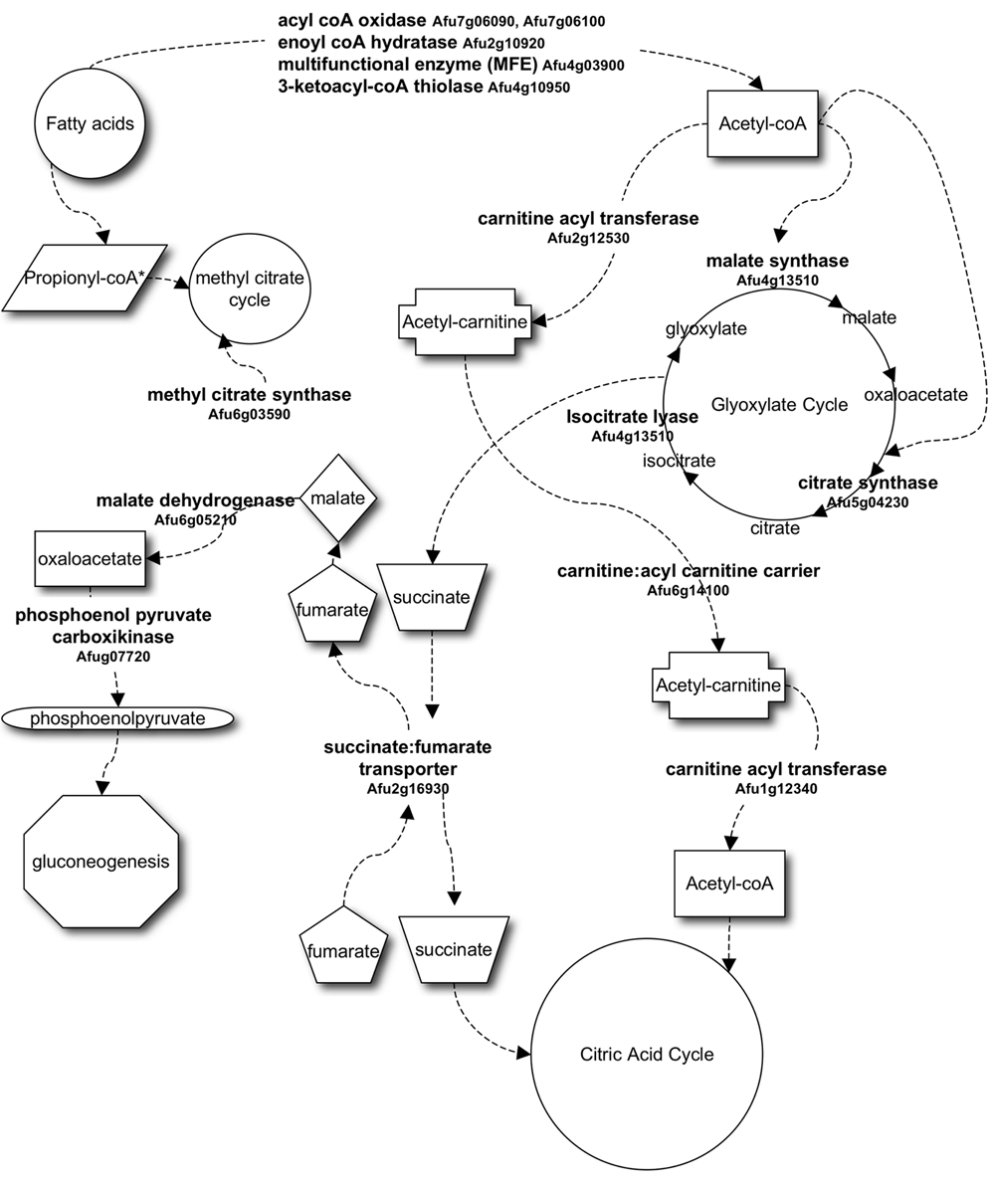
Proposed pathway for utilization of fatty acids in conidia exposed to neutrophils. The pathway was proposed based on the genes that were up-regulated in conidia. The locus ID and the predicted functions of these genes are listed (bold). The fatty acid molecules are degraded via beta-oxidation to produce acetyl-coA units. Two molecules of acetyl-coA are used in the glyoxylate cycle. The malate and oxaloacetate produced in the glyoxylate cycle can participate in gluconeogenesis and the succinate can be used to replenish the citric acid cycle. Alternatively, the acetyl-coA can be transported to mitochondria as acetyl-carnitine and enter the citric acid cycle. The fumarate produced in the citric acid cycle can then be used for gluconeogenesis. *Propionyl-coA is a secondary product of beta-oxidation of fatty acids with even numbers of carbon.

Based on the transcriptome changes, we hereby propose a putative pathway for carbon metabolism in conidia exposed to neutrophils (Fig. 8). The beta-oxidation of fatty acids occurs in cycles that release acetyl units. The acetyl units can either enter the glyoxylate cycle and/or be transported to the mitochondria. An increase in the expression of the isocitrate lyase gene, a key enzyme of the glyoxylate cycle, and genes that encode acetyl-carnitine transferases that transport acetyl units into mitochondria, suggests that both mechanisms may take place in conidia upon phagocytosis by neutrophils. The succinate produced in the glyoxylate cycle can subsequently be transported to the mitochondria to replenish the citric acid cycle. The up-regulation of the gene encoding a putative succinate-fumarate carrier (ACR1) suggests that the enhanced transport of succinate to mitochondria may also occur. A similar mechanism has been described in *S. cerevisiae* where acetyl-coA enters the peroxisomal glyoxylate cycle to produce succinate, which is then transported to the mitochondria via the succinate-fumarate transporter Acr1p [35], [36]. Multiple enzymatic steps can transform the co-transported fumarate into phosphoenolpyruvate, which then can be used for gluconeogenesis. Interestingly, our array data shows that one of the conidial genes exhibiting the highest transcriptional increase upon exposure to neutrophils encodes a formate dehydrogenase. It has been suggested that formate is an indirect product of the glyoxylate cycle and that formate dehydrogenase, Fdh1p, may be involved in the detoxification of formate in *C. albicans* upon phagocytosis by macrophages [37]. It is possible that the *fdh* gene identified in our array study has the same function as that in *C. albicans*.

Previous investigations reported that isocitrate lyase activity was present in germinating conidia of *A. fumigatus* upon phagocytosis by mouse macrophages; however, this enzyme was neither essential for utilization of fatty acids nor for virulence in a murine model suggesting that the glyoxylate cycle is not essential for the pathobiology of this fungus [38], [39]. In contrast, the glyoxylate cycle was shown to be important for virulence of the opportunistic yeast pathogen *Candida albicans* [40]. One hypothesis for the difference found between these two fungi is that *A. fumigatus* may not depend exclusively on the glyoxylate cycle to utilize the acetate units generated by the beta-oxidation of fatty acids. Because our findings suggesting the activation of fatty acid catabolism and glyoxylate cycle in conidia exposed to neutrophils were similar to that observed in *C. albicans* upon phagocytosis by neutrophils and macrophages [30], [33], [37], we investigated the importance of fatty acid catabolism in *A. fumigatus* by deleting the genes *farA* and *farB*. In *A. nidulans,* these genes encode proteins containing Zn_2_-Cys_6_ which are predicted to regulate the genes involved in fatty acid beta-oxidation, transport of acetyl-coA between mitochondria and peroxisomes and the glyoxylate cycle [23]. Deletion of *farA* eliminated the induction of a number of genes by both short and long-chain fatty acids, whereas deletion of *farB* only eliminated the induction by short-chain fatty acids [23]. This study with *A. fumigatus* shows that the deletion of *farA* affects induction of the genes *mfp*, *pex11* and *icl* by long-chain fatty acids and impairs growth in FA but not in glycerol. Deletion of *farB*, however, did not significantly affect the growth in FA or abolish the induction of genes *mfp*, *pex11* and *icl*. Thus, similar to *A. nidulans* [23], the induction of these genes by long-chain fatty acids appears to be mainly dependent on *farA* but not *farB* in *A. fumigatus*. Interestingly, growth of the *farB* deletant in glycerol was comparable to the wild type strain, suggesting that deletion of *farB* did not affect the ability to utilize short-chain molecules such as glycerol. One possibility is that glycerol might be metabolized by a route independent of *farA* or *farB*. Whether the catabolism of short-chain fatty acids, for instance butyrate or hexanoate, is affected by the deletion of *farA* or *farB* is unknown. Although growth assays showed that *farA* is important in the utilization of long-chain fatty acids as the main carbon source during glucose starvation, conidia of the *farA* deletant were no more susceptible to killing by neutrophils than the wild type conidia. Considering that *A. fumigatus* is a ubiquitous and resilient fungus, it is likely that if carbohydrates and fatty acids are not available, conidia might resort to other available nutrients for survival in neutrophils. Thus, it is plausible that the observed changes related to fatty acid catabolism in our array studies is only part of a complex mechanism used by the fungus to survive the neutrophil attack.

To further investigate the molecular mechanisms associated with the response of *A. fumigatus* to neutrophils, we searched for the genes differentially expressed in conidia and hyphae upon exposure to neutrophils. Our findings showed that genes encoding the catalases *cat1* and *cat2*, a manganese superoxide dismutase (Afu1g14550), a glutathione peroxidase (Afu3g12270) and a thioredoxin reductase (Afu4g12990) were up-regulated in hyphae exposed to normal, but not CGD neutrophils (Fig. 6 and Table 5). In contrast, the mRNA levels of both *cat1* and *cat2* and two manganese superoxide dismutases (Afu4g11580 and Afu1g14550) were increased in conidia exposed to normal as well as CGD neutrophils (Fig. 6 and Table 5). An explanation to this difference may be that up-regulation of the two catalases and one of the manganese superoxide dismutases is a hyphal response specific to normal neutrophils whereas in conidia these genes are up-regulated as part of a general stress response. Another possibility is that since neutrophils from individuals with CGD are not always completely defective in producing ROS [6], [41], even small amounts of ROS may be sufficient to elicit an oxidative stress response in conidia phagocytized by neutrophils and sequestered inside vacuoles. Hyphae, however, are larger structures and are not completely engulfed by neutrophils; and therefore, higher concentrations of ROS would be required to elicit a similar response. Similar profiles of gene expression were observed in *C. albicans* exposed to neutrophils [33]. Genes encoding enzymes with catalytic activity such as catalase, glutathione-peroxidase, thioredoxin reductase and superoxide dismutase were found to be up-regulated in *C. albicans* upon interaction with neutrophils. Fradin et al [33] suggested that these genes are likely to be involved in the detoxification of ROS. The role of catalases in *A. fumigatus* pathobiology has been assessed in other studies and it was shown that these enzymes are not essential for fungal virulence [27], [42]. Another study using CGD mice showed that catalases are not required for virulence of *A. nidulans*, the second most common *Aspergillus* species to cause infection in CGD patients [43].

The up-regulation of the metalloreductase and the ferric reductase genes suggests an activation of the reductive system of iron transport. The gene encoding the metalloreductase is predicted to have oxidoreductase activity and is involved in the transport of transition metal ions like iron. In the *S. cerevisiae* model of high-affinity reductive iron transport, the ferric ion is reduced to ferrous ions by the ferric reductases Fre1p and Fre2p [44], [45], [46], [47]. The ferrous ions are then reoxidized by the copper-dependent oxidase Fet3p and transported into the cytoplasm by the iron permease Ftr1p or transported directly by the low-affinity Fet4p. The Fet3p catalytic activity depends on copper ions, which are transported into the cells via Ctr1p [48], [49]. Our array data did not show differential mRNA levels of genes predicted to encode typical copper-dependent oxidases or iron permeases, however, two putative copper transporters were found to be up-regulated. It is conceivable that the up-regulation of these genes is related to the requirement of copper to activate oxidases involved in iron assimilation. In light of the fact that lactoferrin released by neutrophils reduced conidial growth via iron-chelation [4] it is possible that an increase in the mRNA levels of genes involved in iron/copper transport reflect an attempt of the fungal cells to augment iron assimilation. However, experiments with the deletion mutants T9 and T10, both lacking the genes encoding a metalloreductase, a copper transporter and a GPI-anchored protein (Afu6g02800-02820), showed no reduction in growth on iron-limited media (data not shown), suggesting that these genes are not essential for iron assimilation. In fact, it has been reported that assimilation of iron by *A. fumigatus* occurs mainly through siderophores and not reductive systems [50], [51], [52]. Since the up-regulation of these genes is more pronounced in fungal cells exposed to normal neutrophils, it is conceivable that iron/copper assimilation is required as part of a oxidative stress response rather than iron homeostasis. Although T9 and T10 did not show increased susceptibility to hydrogen peroxide, assays with menadione suggested that the three genes are involved in the resistance to the oxidative agent menadione, likely to the superoxide ion generated by this chemical. It is possible that the difference observed in the growth assays with hydrogen peroxide and menadione is due to the mechanism of ROS generation. The connection between iron transport and oxidative stress has previously been described in *S. cerevisiae* [53]. A mutant with deletion of the genes *aft1* and *aft2* that regulate the transcription of high-affinity iron transport genes was observed to be hypersensitive to hydrogen peroxide. It was suggested that these genes have overlapping roles in the control of iron-regulated pathways connected to oxidative stress resistance in yeast [53]. Although the mutants T9 and T10 were less resistant than the wild type to oxidative stress, they were not less resistant to normal neutrophils suggesting that other genes besides the three deleted genes (Afu02800-02820) are required for hyphae to cope with neutrophil attack.

Our findings demonstrate that genes associated with oxidative stress and iron/copper assimilation are differentially regulated in *A. fumigatus* in response to human neutrophils. Interestingly, conidia exposed to neutrophils were found to reprogram their metabolism in a manner similar to their adjustments to glucose-deprivation. The causes and consequences of this metabolic shift remain to be explored further. In vitro testing of the role of some of the differentially expressed genes demonstrated phenotypes in oxidative stress response and fatty acid metabolism. While further studies using in vivo system are required to determine if any of these mutants are less able to establish infections in animals, the identification of fungal genes differentially regulated in response to human immune system attack provided information pertaining to the factors important for fungal survival in the host environment. Characterization of other genes that showed significant differential expression in conidia and hyphae in response to human neutrophils, such as the ones encoding the fungal specific transcription factor (Afu5g02270) and a hypothetical protein (Afu3g02260) may provide important clues as to their roles in *A. fumigatus* pathogenesis.

## Supporting Information

Table S1 List of the genes up-regulated in conidia shown in Figure 2
(0.16 MB DOC)
