# Supplementary material for: Genes Differentially Expressed in Conidia and Hyphae of Aspergillus fumigatus upon Exposure to Human Neutrophils
Source: PLoS One. 2008 Jul 9;3(7):e2655. doi: 10.1371/journal.pone.0002655 (PMC2481287; doi:10.1371/journal.pone.0002655)
Supplement: Table S1 — List of the genes up-regulated in conidia shown in Figure 2 (0.16 MB DOC) [file pone.0002655.s001.pdf]

Table S1. List of the genes up-regulated in conidia shown in Figure 2

PubLocus ID	protein name	N1xH	N2xH	N3xH	C1xH	C2xH	C3xH	N4xS	N5xS	N6xS	N7xS	C4xS	C5xS	C6xS
Afu1g00410	C6 transcription factor, putative	0.33	0.85	0.32	-0.15	0.02	0.10	2.43	3.13	1.79	3.55	2.34	2.37	3.08
Afu1g00450	N-acetylglucosamine-6-phosphate deacetylase (NagA), putative	0.32	0.40	0.54	NaN	0.34	0.02	1.56	0.96	1.79	1.76	2.76	2.32	2.91
Afu1g00480	glucosamine-6-phosphate deaminase, putative	0.68	1.01	0.58	-0.07	0.44	-0.19	1.24	1.19	1.26	1.34	2.33	1.69	2.09
Afu1g01600	deoxyribodipyrimidine photolyase	0.02	0.01	0.17	0.02	0.16	0.59	1.44	1.44	0.95	2.23	1.22	1.03	1.96
Afu1g01610	hypothetical protein	0.16	0.24	0.53	0.13	0.07	0.30	2.37	2.88	1.80	3.44	2.31	2.09	3.19
Afu1g02140	glycogen debranching enzyme, putative	-0.18	0.36	0.29	0.22	0.19	0.30	1.35	1.99	1.66	2.53	1.90	1.36	2.09
Afu1g02540	EF hand domain protein	0.01	0.27	0.16	0.27	0.29	0.48	0.66	1.06	0.81	0.98	0.94	0.91	0.94
Afu1g02820	NADH-quinone oxidoreductase, putative	0.22	0.49	0.01	NaN	-0.16	0.08	1.45	1.00	1.01	2.22	1.35	0.92	2.32
Afu1g02990	WD repeat-containing protein	-0.14	0.15	0.34	0.26	0.04	0.03	0.79	0.97	0.74	NaN	0.77	0.89	1.64
Afu1g03690	conserved hypothetical protein	0.03	0.20	-0.16	NaN	0.38	0.03	1.21	1.13	1.03	1.35	1.14	0.76	1.45
Afu1g04620	alcohol dehydrogenase, zinc-containing, putative	0.07	0.12	-0.06	0.22	0.18	1.44	1.73	2.08	1.95	2.39	1.99	1.50	2.23
Afu1g04670	PSP1 domain protein	-0.10	-0.01	-0.15	0.25	0.06	0.41	1.26	1.36	1.28	1.56	1.47	1.14	2.18
Afu1g04780	peroxisomal ABC transporter (PXA1), putative	0.15	0.49	-0.30	0.08	0.02	-0.11	1.17	2.01	1.38	1.44	1.83	1.13	1.73
Afu1g05980	AMP-binding domain protein, putative	-0.15	0.00	-0.01	-0.12	0.00	0.16	0.76	0.77	0.89	0.72	1.06	0.71	0.98
Afu1g06670	hypothetical protein	0.18	0.05	-0.94	0.17	-0.14	0.55	1.25	1.39	1.77	2.02	1.50	1.47	2.41
Afu1g06820	conserved hypothetical protein	-0.20	0.08	0.43	0.29	0.08	0.21	0.51	0.82	0.61	0.96	0.44	0.76	0.91
Afu1g07380	NADH-dependent glutamate synthase (GLT1), putative	0.02	0.51	0.08	-0.19	0.09	-0.47	1.05	1.12	1.01	1.40	0.78	1.13	1.40
Afu1g07540	proteasome regulatory particle subunit (RpnH), putative	-0.32	0.21	-0.09	0.17	0.19	0.11	0.79	0.57	0.49	0.94	0.94	0.75	1.02
Afu1g09030	hypothetical protein	0.58	0.25	-0.26	NaN	-0.02	0.31	2.69	2.73	2.92	3.29	2.94	2.74	3.40
Afu1g09470	aminotransferase, class V, putative	-0.21	-0.03	-0.02	0.35	0.17	0.13	0.69	0.56	0.50	1.25	0.76	0.62	1.39
Afu1g09930	glycerol dehydrogenase (GCY1), putative	-0.30	0.44	0.81	0.14	0.35	0.94	2.08	2.55	1.86	2.42	2.04	1.72	2.23
Afu1g10350	phosphoglycerate kinase PgkA, putative	-0.37	-0.39	-0.53	0.10	-0.05	0.29	0.66	0.80	0.79	0.73	1.01	0.99	0.82
Afu1g11480	hypothetical protein	-0.18	0.01	-0.32	0.21	-0.27	-0.37	1.00	1.29	1.31	1.16	0.89	1.71	0.77
Afu1g12650	3-ketoacyl-CoA ketothiolase (Kat1), putative	0.02	0.38	-0.13	0.19	0.14	-0.15	1.55	2.26	2.04	1.44	1.73	1.28	1.37
Afu1g12910	ABC fatty acid transporter, putative	0.05	0.36	-0.18	0.24	0.38	0.13	1.05	1.21	1.25	0.89	1.30	1.14	1.22
Afu1g12920	glycogen phosphorylase 1; possible glycogen phosphorylase	-0.53	0.38	-0.90	0.19	0.31	0.54	2.49	2.92	2.54	2.37	2.61	2.71	2.80
Afu1g12930	G protein complex alpha subunit GpaB	0.14	0.27	0.21	0.07	-0.15	0.46	0.72	0.78	0.45	1.11	0.74	0.57	1.13
Afu1g13370	aldo-keto reductase (AKR7), putative	0.01	0.62	0.33	0.08	0.19	0.23	2.25	2.13	1.87	2.38	2.37	1.82	2.42
Afu1g13510	C6 transcription factor (FacB), putative	-0.56	0.96	0.03	-0.20	-0.01	-0.14	2.84	2.98	2.23	3.23	2.77	2.54	3.34
Afu1g13520	SET domain protein	0.17	-0.32	-0.11	0.13	-0.09	-0.11	0.82	0.78	0.63	1.00	0.93	0.72	0.78
Afu1g14080	integral membrane protein PTH11, putative	0.19	0.31	-0.15	0.22	0.09	-0.03	1.64	2.54	1.92	1.68	2.36	1.99	1.96
Afu1g14850	acyl-CoA dehydrogenase, putative	-0.06	0.65	-0.02	0.12	0.04	-0.16	NaN	1.72	1.65	1.34	1.33	0.95	1.40
Afu1g15590	succinate dehydrogenase subunit CybS, putative	-0.26	-0.32	-0.01	-0.38	-0.14	-0.52	0.50	1.01	0.23	0.81	0.77	0.73	0.89
Afu2g01010	myo-inositol-phosphate synthase, putative	0.09	0.24	-0.24	0.18	0.35	0.49	0.83	0.86	0.76	1.52	1.00	0.64	1.47

Afu2g01040	formaldehyde dehydrogenase	-0.15	0.01	0.24	-0.08	0.17	-0.39	0.77	1.51	1.43	0.71	0.91	0.92	0.70
Afu2g01220	GTP cyclohydrolase II, putative	-0.07	0.40	-0.30	0.10	0.10	0.28	1.04	1.22	1.41	1.17	1.20	0.44	1.61
Afu2g02310	cortical patch protein SUR7, putative	0.19	0.45	1.24	0.16	0.04	1.88	2.54	1.47	1.72	4.14	2.21	1.70	3.83
Afu2g02630	hypothetical protein	-0.12	0.38	-0.05	0.16	0.01	0.58	0.61	0.54	0.58	0.93	0.91	0.56	0.90
Afu2g03860	plasma membrane low affinity zinc ion transporter, putative	-0.01	-0.01	-0.33	-0.43	-0.01	-0.13	1.56	1.28	0.82	1.66	1.80	1.41	1.68
Afu2g04080	GPR/FUN34 family protein	-0.81	-1.16	-1.19	-0.38	-0.93	-0.33	3.12	2.86	2.42	3.47	3.42	2.69	3.38
Afu2g05250	transcription factor RfeD, putative	0.22	0.16	0.38	0.32	0.25	0.12	0.88	1.00	0.84	1.19	0.92	1.13	1.24
Afu2g05840	MFS multidrug transporter, putative	NaN	-0.19	-0.06	-0.12	-0.12	-0.14	1.32	0.86	0.85	1.84	1.35	1.09	1.64
Afu2g07420	fimbrin	-0.07	0.43	0.20	0.02	0.38	0.20	0.52	0.73	0.50	1.08	0.55	0.87	1.11
Afu2g08040	C6 finger domain protein, putative	-0.07	-0.01	-0.16	0.09	0.02	-0.06	1.24	0.98	0.92	0.79	1.15	0.87	0.82
Afu2g08900	voltage-gated chloride channel (ClcA), putative	NaN	-0.38	-0.09	0.11	-0.02	0.08	0.74	0.82	0.79	1.32	0.88	0.95	1.19
Afu2g08920	GDSL Lipase/Acylhydrolase family protein	-0.03	-0.10	-0.15	-0.06	0.17	0.24	0.93	0.86	0.68	0.88	1.06	0.58	1.01
Afu2g08950	isochorismatase family hydrolase, putative	NaN	0.11	0.13	0.21	0.34	0.15	1.48	1.56	1.57	1.89	1.46	1.15	1.74
Afu2g09640	RING finger domain protein, putative	0.07	0.63	0.40	0.26	0.00	0.34	1.90	2.40	1.51	2.48	1.50	1.90	2.34
Afu2g09650	aspartate transaminase, putative	0.42	0.63	0.29	0.15	0.07	0.32	1.42	1.46	1.13	1.98	1.44	1.58	1.67
Afu2g09850	oxidoreductase, 2-nitropropane dioxygenase family, putative	-0.11	0.55	-0.43	-0.03	0.15	0.00	0.88	1.42	1.27	0.94	1.61	0.53	0.77
Afu2g10150	peroxisome biosynthesis protein (PAS1/Peroxin-1), putative	0.08	0.20	0.12	0.05	0.05	0.11	0.86	1.07	0.92	0.98	1.17	0.85	1.02
Afu2g10690	MFS phosphate transporter, putative	-0.66	0.18	0.01	-0.01	-0.19	0.14	0.78	0.73	0.41	1.07	0.61	0.91	1.02
Afu2g10910	MFS alpha-glucoside transporter, putative	-0.14	0.03	-0.01	-0.26	0.12	-0.18	0.91	0.67	0.79	0.93	0.86	0.66	1.66
Afu2g10920	enoyl-CoA hydratase/isomerase family protein	-0.06	0.37	0.05	0.14	0.24	0.16	0.94	1.32	0.96	0.73	2.01	0.70	1.05
Afu2g11340	ML domain protein, putative	-0.10	0.86	-0.01	0.20	-0.07	0.21	1.19	1.15	1.25	1.58	1.42	1.04	1.43
Afu2g11570	F-box domain protein	-0.18	0.43	0.01	0.10	0.05	0.58	1.60	1.36	1.41	2.02	1.56	NaN	1.73
Afu2g11900	pyruvate dehydrogenase kinase	NaN	0.74	0.02	0.35	-0.09	0.13	3.46	2.97	2.48	4.23	3.31	3.25	4.05
Afu2g12200	cAMP-dependent protein kinase catalytic subunit PkaC1	-0.42	-0.31	0.13	-0.10	-0.13	0.02	0.62	0.67	0.39	1.24	0.55	0.64	1.21
Afu2g12330	conserved hypothetical protein	-0.11	0.27	-0.04	0.13	0.00	0.01	1.17	1.03	0.99	1.30	1.31	0.97	1.38
Afu2g12450	hydroxymethylglutaryl-CoA lyase	-0.09	0.15	-0.16	0.20	0.09	0.42	1.29	1.59	1.78	1.36	1.41	1.29	1.71
Afu2g12530	carnitine acetyl transferase	0.14	0.19	-0.02	0.26	-0.01	-0.24	2.18	2.80	2.34	3.01	3.32	1.80	2.46
Afu2g13580	conserved hypothetical protein	-0.12	0.79	0.13	0.27	0.47	0.33	1.47	1.67	1.72	1.53	1.52	1.82	1.38
Afu2g13590	hypothetical protein	-0.13	0.95	0.07	0.10	0.39	0.82	1.61	2.06	1.95	1.78	1.91	1.96	1.80
Afu2g14160	autophagy protein Atg20, putative	-0.15	0.18	0.20	0.04	0.10	0.49	0.56	0.85	0.72	1.01	0.75	NaN	1.11
Afu2g14590	MFS monosaccharide transporter, putative	-0.54	1.10	1.00	0.06	0.82	0.61	1.46	2.09	1.42	1.81	1.59	1.50	1.99
Afu2g15960	nucleotide binding protein Nbp35, putative	NaN	0.42	0.23	-0.18	0.03	-0.20	0.81	1.12	0.86	1.11	0.75	0.91	1.07
Afu2g16320	MFS multidrug transporter, putative	0.07	0.22	-0.02	-0.32	0.14	-0.10	0.89	0.96	0.27	1.53	1.23	0.56	1.19
Afu2g16930	succinate:fumarate antiporter (Acr1), putative	0.21	0.36	-0.21	-0.48	-0.08	0.03	4.18	3.62	3.19	4.55	4.17	2.71	4.55
Afu2g16950	hypothetical protein	-0.19	0.07	-0.14	0.26	-0.03	0.08	1.07	1.28	1.03	1.10	1.17	0.82	1.13
Afu2g17220	C2H2 transcription factor (AmdX), putative	0.21	0.14	-0.16	0.35	0.13	0.23	0.77	1.33	1.33	1.27	1.06	1.08	1.60
Afu3g00810	cholestenol delta-isomerase, putative	0.04	0.06	-0.25	-0.03	0.08	1.09	0.26	0.18	0.30	0.69	2.27	-0.23	1.41
Afu3g00840	FAD-dependent oxygenase, putative	-0.31	-0.44	-0.52	-0.11	-0.26	0.42	0.99	0.93	0.91	1.57	0.73	0.59	1.63
Afu3g00850	hypothetical protein	-0.09	0.23	-0.48	-0.11	-0.26	0.13	0.69	0.56	0.64	1.25	0.53	0.22	1.37

Afu3g00860	hypothetical protein	-0.33	-0.28	-0.56	-0.07	-0.27	0.29	1.01	0.87	0.92	1.77	0.86	0.74	1.72
Afu3g00900	alpha-amylase, putative	-0.29	-0.08	-0.12	0.00	0.06	0.33	0.60	0.93	0.45	0.94	1.08	0.45	0.95
Afu3g01330	class II aldolase/adducin domain protein	NaN	0.32	-0.28	-0.05	0.16	-0.11	0.66	0.71	0.72	0.75	1.21	0.28	0.63
Afu3g02000	C6 transcription factor, putative	0.13	0.71	-0.35	0.05	0.22	0.45	1.47	0.85	1.02	2.65	1.53	1.08	2.53
Afu3g02280	alpha,alpha-trehalose glucohydrolase, putative	0.46	1.13	1.00	0.29	0.66	0.20	3.68	3.89	3.86	4.20	3.45	3.52	3.69
Afu3g02890	MFS sugar transporter, putative	0.34	-0.29	-0.14	-0.20	0.08	-0.15	1.42	2.27	2.24	0.91	1.51	3.03	1.04
Afu3g02900	oxidoreductase, short chain dehydrogenase/reductase family	0.17	0.32	0.17	-0.03	0.02	-0.11	1.53	2.15	2.14	0.85	1.23	2.40	1.00
Afu3g02940	allergen, putative	-0.05	0.02	-0.10	-0.01	0.25	0.28	0.85	0.67	0.40	1.49	0.71	0.29	1.33
Afu3g03700	MFS sugar transporter, putative	0.57	-0.16	-0.18	-0.32	-0.01	-0.54	1.48	2.08	1.66	1.08	1.54	1.92	1.37
Afu3g04150	mitochondrial enoyl reductase, putative	0.04	0.31	0.55	0.15	0.04	0.63	1.75	1.62	1.00	2.36	1.51	1.40	2.18
Afu3g05820	ZZ type zinc finger domain protein	-0.01	0.89	0.23	0.07	0.57	0.62	1.07	1.61	1.13	1.74	1.96	0.89	1.70
Afu3g06050	fungal specific transcription factor, putative	-0.15	0.38	0.13	0.11	0.08	-0.06	0.88	1.57	1.51	1.37	1.35	1.23	1.37
Afu3g06070	histone H1	0.33	0.29	0.05	0.23	-0.01	0.10	0.49	1.13	0.78	1.11	0.88	1.35	1.39
Afu3g06540	3'-phosphoadenosine-5'-phosphosulfate reductase	0.47	1.27	0.74	-0.01	0.01	-0.15	1.30	1.85	1.32	2.19	0.72	1.09	1.65
Afu3g06660	NIPSNAP family protein	-0.17	0.07	0.29	NaN	0.07	0.36	1.90	1.89	1.93	2.18	1.47	1.20	2.19
Afu3g06670	pyridoxamine phosphate oxidase, putative	-0.29	-0.18	0.25	0.16	0.08	0.37	1.00	1.34	1.07	1.79	1.08	1.13	1.80
Afu3g07180	pantothenate kinase, putative	0.03	-0.33	-0.39	0.13	-0.57	-0.17	0.84	0.57	0.30	1.20	0.38	0.47	1.14
Afu3g07810	succinate dehydrogenase, flavoprotein subunit	-0.23	-0.34	0.11	-0.21	0.05	-0.11	0.74	1.03	0.23	1.46	0.93	0.82	1.34
Afu3g08240	conserved hypothetical protein	-0.11	0.39	-0.20	-0.05	-0.21	-0.06	0.44	0.44	0.61	0.88	0.77	1.01	0.72
Afu3g08650	C1 tetrahydrofolate synthase, putative	-0.38	0.17	0.03	0.22	0.06	0.15	0.70	0.41	0.32	0.97	0.66	0.65	0.97
Afu3g08900	tubulin-specific chaperone c, putative	0.43	0.25	0.43	0.21	0.05	0.29	1.44	1.66	1.64	2.86	1.57	1.16	2.91
Afu3g09390	AMMECR1 family protein	0.00	0.10	-0.07	0.24	0.03	0.21	0.57	0.89	0.75	0.89	0.49	0.64	NaN
Afu3g10000	cAMP-dependent protein kinase regulatory subunit PkaR	-0.13	0.13	0.54	0.17	0.26	0.42	0.88	0.96	0.70	1.38	0.70	0.93	1.14
Afu3g10300	galactokinase	-0.19	0.21	-0.12	-0.04	0.43	0.10	0.98	1.43	1.39	0.95	1.10	1.24	0.86
Afu3g10530	protein serine/threonine kinase (Ran1), putative	-0.02	0.73	-0.49	0.32	0.59	0.07	1.18	1.40	1.60	1.61	1.54	1.40	2.33
Afu3g11400	aspartic endopeptidase Pep2	0.78	0.92	0.20	0.21	0.23	0.78	2.15	2.89	2.55	3.50	2.69	2.21	3.33
Afu3g11830	phosphoglucomutase PgmA	-0.06	0.05	-0.17	0.20	0.12	0.32	0.32	0.78	0.53	0.71	0.62	0.76	0.91
Afu3g12770	nucleoside-diphosphate-sugar epimerase, putative	0.15	0.07	-0.13	-0.10	0.06	0.02	0.95	1.26	1.16	0.49	0.70	0.90	0.83
Afu3g12790	conserved glutamic acid-rich protein	-0.31	0.28	0.03	0.02	0.16	0.46	0.66	1.19	0.72	0.75	1.18	1.02	0.61
Afu3g12890	C6 transcription factor (GliZ), putative	0.10	0.01	0.04	0.24	0.22	-0.12	0.62	0.55	0.81	1.18	0.63	0.56	1.32
Afu3g13080	hypothetical protein	0.27	0.98	-0.33	-0.09	0.03	0.17	1.49	1.09	1.02	2.03	1.39	0.66	1.93
Afu4g00150	MFS maltose transporter, putative	0.16	0.26	-0.14	-0.05	0.27	-0.10	1.32	1.68	1.75	1.20	1.95	1.98	1.43
Afu4g00290	succinyl-CoA synthetase beta subunit, putative	-0.32	0.02	-0.08	-0.16	-0.05	0.04	0.33	0.72	0.52	1.21	0.85	0.35	1.72
Afu4g00800	MFS monosaccharide transporter, putative	-0.37	1.08	0.26	-0.09	0.86	0.19	2.64	3.75	2.80	2.82	2.74	2.56	2.73
Afu4g05900	conserved hypothetical protein	0.20	1.25	1.91	0.07	-0.05	1.30	3.01	2.89	3.07	4.20	2.53	2.53	4.17
Afu4g06190	fungal specific transcription factor, putative	-0.06	0.27	-0.11	-0.04	0.08	-0.06	0.69	1.09	0.59	0.62	0.91	1.03	0.91
Afu4g06380	sterol carrier protein, putative	0.02	0.14	-0.22	-0.13	0.02	-0.09	1.39	1.64	1.42	1.49	1.08	0.54	1.42
Afu4g06420	fungal specific transcription factor, putative	-0.07	-0.07	0.27	0.00	0.11	0.14	0.77	1.39	0.91	0.73	1.46	0.68	0.79
Afu4g07030	hypothetical protein	-0.30	-0.08	-0.33	0.43	-0.58	0.57	0.48	1.31	1.00	1.63	1.19	1.58	1.98

Afu4g07040	aspartic-type endopeptidase (CtsD), putative	-0.54	-0.50	-0.81	0.09	-0.58	0.28	0.72	0.95	0.81	1.14	1.02	0.90	1.37
Afu4g08240	alcohol dehydrogenase, zinc-containing	-0.03	0.82	0.85	0.06	-0.01	0.31	2.10	2.01	2.06	3.14	1.80	1.05	3.27
Afu4g08440	Patatin-like serine hydrolase, putative	NaN	0.30	0.11	0.10	0.10	-0.03	0.83	1.30	1.12	1.38	0.71	1.19	1.28
Afu4g08490	acyl-CoA dehydrogenase, putative	0.25	0.22	-0.07	0.04	-0.07	-0.27	0.96	0.67	0.34	0.87	0.97	0.17	0.87
Afu4g08580	antioxidant protein LsfA	0.31	1.12	0.06	0.04	0.52	0.25	2.19	3.77	2.52	2.86	2.30	2.54	2.21
Afu4g08710	short chain dehydrogenase, putative	0.37	0.16	-0.84	0.06	0.37	0.26	0.98	0.91	0.88	1.44	1.07	1.09	2.25
Afu4g09110	cytochrome c peroxidase, putative	0.18	0.45	-0.11	NaN	-0.06	-0.04	2.28	3.33	2.88	2.65	1.93	2.17	1.95
Afu4g10120	C6 finger domain protein, putative	-0.51	-0.85	-0.87	-0.06	-0.14	0.42	0.59	0.40	0.61	0.39	0.94	0.43	1.19
Afu4g10340	C6 finger domain protein, putative	0.06	-0.25	-0.41	0.14	-0.44	0.05	0.70	0.50	0.65	0.84	0.62	NaN	0.80
Afu4g10410	aspartate aminotransferase, putative	-0.08	0.36	-0.18	0.26	0.13	0.50	0.98	0.82	0.85	1.23	1.30	1.04	1.01
Afu4g10950	3-ketoacyl-coA thiolase peroxisomal A precursor	-0.49	0.19	-0.40	0.23	-0.35	-0.61	2.28	2.56	2.27	1.62	2.30	1.03	1.77
Afu4g11080	acetyl-coenzyme a synthetase	-0.57	-0.36	-0.38	0.21	-0.39	-0.22	4.02	4.07	3.33	4.83	4.07	3.62	4.77
Afu4g11540	glycerol kinase, putative	-0.13	0.12	0.58	0.14	0.20	0.15	1.51	1.89	1.23	1.98	0.89	1.32	1.90
Afu4g11580	Mn superoxide dismutase (SodB), putative	0.08	0.13	0.02	-0.09	-0.04	0.04	1.24	1.09	1.09	1.27	1.04	0.94	1.42
Afu4g11710	oxidoreductase, zinc-binding dehydrogenase family superfamily	0.20	0.22	0.17	-0.19	0.04	0.05	0.81	1.02	0.58	1.26	0.56	0.61	0.85
Afu4g12010	2-oxo acid dehydrogenases acyltransferase, putative	0.27	0.66	0.05	0.20	0.23	0.05	0.54	0.96	0.99	0.56	0.74	0.35	1.34
Afu4g12870	methylmalonate-semialdehyde dehydrogenase, putative	-0.22	0.79	0.41	0.23	0.44	0.29	2.08	2.60	2.20	1.98	2.22	2.13	2.32
Afu4g12880	hypothetical protein	0.05	0.40	-0.07	0.12	-0.03	0.11	0.85	1.25	0.66	0.91	1.31	0.61	0.67
Afu4g12950	vacuolar targeting protein Atg24, putative	-0.23	-0.07	0.24	0.14	0.11	0.17	0.59	0.73	0.71	1.07	0.60	0.50	1.13
Afu4g13080	MFS monosaccharide transporter, putative	0.11	0.19	-0.46	0.07	0.66	0.86	3.08	2.79	2.63	3.49	2.53	2.74	3.24
Afu4g13500	aldehyde dehydrogenase, putative	0.08	0.07	-0.13	0.16	-0.13	0.04	0.82	0.94	0.74	0.87	0.62	0.88	0.83
Afu4g13510	isocitrate lyase	0.23	0.79	0.34	-0.11	-0.17	0.05	4.55	4.24	3.89	5.30	4.99	3.39	5.23
Afu4g13590	hypothetical protein	0.12	0.06	-0.12	0.57	-0.34	0.12	0.89	1.04	1.00	1.59	1.11	0.68	1.37
Afu4g14110	hypothetical protein	-0.06	0.68	0.09	0.05	-0.01	0.57	1.06	1.26	0.93	1.55	1.19	1.02	0.73
Afu5g00310	flavin-containing monooxygenase, putative	0.30	0.21	-0.20	NaN	0.07	0.01	2.60	2.79	2.71	2.59	3.49	2.14	2.99
Afu5g01380	conserved hypothetical protein	-0.39	NaN	0.03	0.09	-0.14	0.36	1.01	0.57	0.53	1.04	0.66	0.52	1.05
Afu5g01800	DNA repair protein rad14	0.26	0.46	0.05	0.20	0.11	0.13	1.16	0.97	0.94	1.81	1.67	0.89	1.69
Afu5g01820	DUF221 domain protein, putative	0.04	0.07	0.01	0.15	0.23	0.22	0.88	1.20	0.50	0.70	1.06	1.04	0.54
Afu5g02350	hydrolase, carbon-nitrogen family, putative	-0.16	0.03	-0.29	-0.14	0.21	0.25	0.93	2.04	1.09	1.15	1.11	1.05	1.02
Afu5g02480	glycogen synthase	0.02	0.40	-0.08	0.31	0.20	0.40	2.73	2.89	2.78	3.01	2.62	2.22	2.94
Afu5g02780	mitochondrial nicotinamide nucleotide transhydrogenase subunit, putative	-0.01	0.03	-0.13	0.25	0.26	0.16	0.95	1.07	1.08	1.25	1.06	1.03	1.35
Afu5g02800	C6 transcription factor, putative	-0.37	-0.39	-0.51	0.21	-0.24	0.06	0.78	0.97	0.85	1.11	1.08	0.85	1.10
Afu5g03740	isopenicillin N-CoA epimerase, putative	0.02	0.05	0.16	0.06	0.07	0.05	0.95	0.79	0.63	1.24	0.87	0.68	1.09
Afu5g04180	hypothetical protein	0.05	-0.03	-0.36	0.12	-0.07	0.18	0.79	1.03	0.86	1.15	1.06	0.50	1.25
Afu5g04250	homocysteine synthase, putative	0.46	1.41	-0.13	-0.04	0.13	0.53	2.86	3.88	3.03	3.95	2.53	2.94	3.50
Afu5g04310	peroxisomal membrane protein Pmp47, putative	-0.17	0.10	-0.31	0.21	-0.43	0.08	1.46	1.02	1.15	1.95	1.40	0.78	1.78
Afu5g07000	NAD binding Rossmann fold oxidoreductase, putative	-0.53	0.08	0.40	0.12	0.30	0.06	1.12	2.35	2.02	2.55	1.10	0.59	2.44
Afu5g07400	phenylacetyl-CoA ligase PclA, putative	-0.07	0.22	-0.37	0.18	0.34	-0.15	1.74	2.16	1.82	1.37	1.81	1.29	1.44
Afu5g08020	HLH DNA binding protein (Penr2), putative	-0.35	-0.09	-0.19	0.09	-0.02	0.05	0.55	0.75	0.69	0.80	0.97	0.73	0.91

Afu5g08440	conserved hypothetical protein	0.00	-0.09	-0.47	0.03	-0.04	-0.01	0.49	0.58	0.58	0.79	0.64	NaN	1.06
Afu5g08470	AMP-binding enzyme, putative	NaN	0.08	-0.30	0.12	0.43	0.02	1.53	2.63	1.88	1.81	1.74	1.07	2.24
Afu5g08910	3-methylcrotonyl-CoA carboxylase subunit alpha (MccA), putative	0.19	0.75	0.04	0.22	0.23	0.18	1.18	1.48	1.81	1.34	1.30	0.64	2.20
Afu5g09210	autophagic serine protease Alp2	0.26	1.59	0.27	0.28	0.35	0.23	1.58	1.63	1.68	1.93	1.68	1.59	1.82
Afu5g09310	Bax Inhibitor family protein	-0.39	-0.05	-0.09	0.19	0.09	0.29	0.51	0.65	0.66	0.99	0.84	NaN	1.21
Afu5g09400	carbonyl reductase, putative	-0.49	-0.18	-0.55	0.04	0.03	-0.12	0.70	0.83	0.71	0.67	0.87	NaN	0.49
Afu5g09860	esterase, putative	-0.20	-0.46	-0.20	0.05	0.25	-0.01	NaN	1.32	1.04	0.51	1.10	1.00	0.63
Afu5g10370	succinate dehydrogenase iron-sulphur protein	0.26	-0.20	0.00	-0.38	-0.16	-0.23	0.80	1.18	0.41	1.55	1.03	0.99	1.33
Afu5g11690	protein tyrosine phosphatase Pps1, putative	0.11	0.10	-0.49	0.32	-0.05	0.21	1.21	2.08	1.41	2.10	1.86	1.77	2.18
Afu5g11750	LON domain serine protease, putative	-0.11	0.11	-0.11	0.05	-0.13	0.17	0.63	0.78	0.94	0.94	0.81	0.38	0.89
Afu5g12840	hydroxyacylglutathione hydrolase, putative	-0.37	NaN	0.17	-0.28	0.23	0.09	1.17	1.63	1.20	1.76	1.34	1.23	1.59
Afu5g13650	hypothetical protein	0.89	0.60	0.26	0.47	0.31	-0.16	2.45	2.07	1.31	2.81	2.40	1.98	2.19
Afu5g13810	transulfuration enzyme family protein, putative	0.21	-0.25	-0.22	0.04	-0.13	-0.40	0.60	1.28	0.55	1.12	0.70	0.95	0.88
Afu5g14210	glucose repressible protein Grg1, putative	0.00	2.04	2.66	0.16	-0.01	2.33	3.38	3.43	2.68	3.34	3.15	2.67	3.36
Afu5g14650	RING finger protein	-0.94	-1.39	-0.77	-0.14	-0.36	-0.13	0.73	0.86	0.34	1.08	0.49	NaN	1.43
Afu6g01940	conserved hypothetical protein	0.03	0.13	0.12	0.07	0.18	0.55	1.39	1.68	1.77	1.70	1.68	1.59	1.76
Afu6g01950	hypothetical protein	0.63	-0.19	-0.27	0.10	0.12	0.29	0.69	0.70	0.44	1.73	0.40	0.63	1.48
Afu6g02030	aminotransferase, putative	NaN	0.58	-0.50	-0.07	0.00	0.26	1.10	0.57	0.49	1.19	1.30	0.59	1.47
Afu6g02860	isocitrate lyase	-0.34	0.69	-0.11	0.06	0.26	0.32	2.27	2.40	2.16	2.09	2.41	1.71	2.46
Afu6g03060	MFS monosaccharide transporter, putative	-0.73	0.13	-0.21	0.15	1.11	0.25	2.22	3.27	2.22	2.32	2.92	2.42	2.72
Afu6g03400	conserved hypothetical protein	0.73	0.24	-0.08	0.50	0.04	2.23	2.31	1.75	1.81	3.91	2.68	0.63	3.62
Afu6g03530	glutamine synthetase	-0.01	-0.21	-0.05	0.23	-0.06	0.05	1.45	1.25	1.07	1.64	1.53	1.27	1.64
Afu6g03590	methylcitrate synthase	-0.24	0.09	-0.07	-0.16	-0.12	0.14	1.81	2.16	1.81	2.17	2.61	1.14	2.31
Afu6g03730	2-methylcitrate dehydratase, putative	-0.02	0.65	-0.10	0.06	0.31	0.25	2.81	2.52	2.26	3.04	2.55	2.13	3.10
Afu6g04270	MFS sugar transporter, putative	-0.49	-0.57	-0.17	-0.14	0.58	0.58	1.20	1.09	1.03	1.27	1.84	1.20	2.20
Afu6g04490	camp independent regulatory protein	0.16	-0.42	-0.51	-0.03	-0.27	-0.25	0.17	0.84	0.18	1.05	0.26	0.94	1.15
Afu6g04920	NAD-dependent formate dehydrogenase AciA/Fdh	0.22	0.46	-0.24	0.13	0.02	1.26	4.88	4.72	4.54	4.48	5.05	3.34	4.79
Afu6g05210	malate dehydrogenase, NAD-dependent	-0.13	0.01	-0.08	-0.04	0.25	-0.19	2.02	1.97	1.61	2.13	1.95	1.95	2.21
Afu6g06500	actin-related protein 2/3 complex subunit 1A, putative	-0.09	0.16	0.00	0.01	0.07	0.19	0.64	0.76	0.58	0.78	0.72	0.73	0.75
Afu6g06830	RNA polymerase II mediator complex subunit Srb5, putative	0.11	-0.09	-0.19	0.14	-0.28	0.51	0.84	0.66	0.56	1.02	0.98	0.77	1.08
Afu6g06840	hypothetical protein	0.41	-0.19	-0.33	0.10	-0.29	0.41	0.65	0.59	0.50	0.99	0.98	0.52	1.04
Afu6g07090	maleylacetate reductase, putative	-0.07	-0.10	-0.32	-0.08	-0.16	-0.04	0.89	1.14	0.75	0.87	0.75	0.93	0.86
Afu6g07280	ABC transporter (Adp1), putative	0.29	0.31	0.07	0.10	0.05	0.11	0.85	0.92	0.81	1.28	0.72	0.61	1.20
Afu6g07720	phosphoenolpyruvate carboxykinase (ATP)	-0.07	0.61	1.60	0.05	-0.32	0.77	3.21	3.16	1.83	4.46	3.56	2.01	4.33
Afu6g07740	peroxisomal biogenesis factor (PEX11), putative	-0.17	0.13	-0.07	0.11	-0.17	-0.06	2.27	2.26	2.03	2.36	2.42	1.61	2.30
Afu6g08930	conserved hypothetical protein	0.12	0.35	0.30	-0.05	0.00	0.32	0.67	0.86	0.77	1.35	1.01	0.27	1.17
Afu6g09980	sphingolipid transporter (Ncr1), putative	-0.01	0.33	0.01	0.25	0.02	-0.06	1.00	1.17	1.08	1.27	1.06	0.73	1.07
Afu6g10040	fructosyl amine: oxygen oxidoreductase	-0.82	-0.50	-0.52	0.04	0.12	-0.10	0.15	1.12	0.30	0.90	0.35	0.46	1.48
Afu6g10080	conserved hypothetical protein	0.04	-0.05	-0.10	0.20	-0.39	0.40	3.21	2.75	2.41	3.95	3.50	2.05	3.41

Afu6g10260	aldehyde reductase (AKR1), putative	-0.24	1.00	0.31	-0.17	0.75	-0.20	2.06	3.06	2.69	1.78	2.44	2.25	1.58
Afu6g10440	hypothetical protein	-0.25	-0.35	0.59	0.14	-0.05	0.38	0.65	1.00	1.09	0.99	0.94	0.71	0.77
Afu6g10720	alpha-ketoglutarate-dependent taurine dioxygenase	0.34	0.11	-0.14	0.09	0.12	0.04	1.53	1.50	1.90	2.62	0.93	1.01	1.66
Afu6g10880	acyl-CoA dehydrogenase, putative	NaN	0.11	0.16	-0.19	-0.22	-0.28	1.01	1.76	1.42	1.13	1.67	0.76	1.15
Afu6g11430	aldehyde dehydrogenase, putative	-0.22	1.16	0.19	-0.07	0.30	-0.31	3.33	3.77	3.23	3.92	3.39	3.20	3.66
Afu6g11950	hypothetical protein	0.23	0.14	0.02	0.23	0.20	0.02	1.45	1.54	1.24	1.90	1.26	1.40	1.52
Afu6g12180	conserved hypothetical protein	0.54	1.68	3.50	0.61	0.58	2.88	2.72	2.68	1.60	4.62	1.96	1.57	4.50
Afu6g12290	PH domain protein	0.08	0.48	0.52	0.23	0.45	0.57	2.01	1.92	1.48	3.24	1.52	1.40	2.94
Afu6g12680	HIT domain protein	-0.48	0.16	-0.20	-0.02	0.27	0.11	0.88	1.34	1.11	0.63	1.24	1.30	0.73
Afu6g12870	ABC iron exporter Atm1, putative	-0.09	-0.24	-0.20	0.30	-0.08	-0.03	0.76	0.67	0.64	0.86	0.66	0.57	0.62
Afu6g12930	mitochondrial aconitate hydratase, putative	0.00	-0.78	-0.34	-0.03	-0.24	-0.19	1.06	1.18	0.43	1.49	1.09	0.62	1.43
Afu6g12950	alpha,alpha-trehalose-phosphate synthase subunit TPS1, putative	0.34	-0.11	-0.12	0.11	-0.11	0.02	0.53	0.82	0.67	0.92	0.94	0.31	0.92
Afu6g13150	hypothetical protein	-0.04	-0.12	-0.12	0.13	-0.01	0.14	0.68	1.02	0.76	0.79	1.41	0.62	0.96
Afu6g13200	autophagy regulatory protein Atg2, putative	0.36	0.51	-0.04	0.31	0.13	0.33	0.92	0.65	0.78	1.32	0.90	0.62	1.31
Afu6g13330	conserved hypothetical protein	0.50	0.98	1.79	0.44	0.26	2.08	2.92	2.35	2.27	4.43	2.57	1.83	4.20
Afu6g13540	carboxypeptidase Y (CpyA), putative	0.51	1.82	0.48	0.16	0.37	0.24	2.01	2.09	2.10	2.78	2.09	1.47	2.54
Afu6g13590	3-isopropylmalate dehydrogenase	-0.07	-0.05	-0.28	0.23	0.06	0.09	1.16	1.45	1.52	0.79	1.34	0.74	1.12
Afu6g14090	CFEM domain protein, putative	-0.23	-0.31	0.17	0.07	0.16	0.47	0.92	0.75	0.39	1.07	1.27	0.79	1.33
Afu6g14100	mitochondrial carnitine:acyl carnitine carrier, putative	-0.44	-0.19	0.11	0.06	0.00	-0.17	2.39	2.14	1.53	2.49	2.42	1.83	2.65
Afu6g14200	acetyl-CoA-acetyltransferase, putative	0.09	0.59	-0.01	-0.17	-0.07	0.02	0.94	0.84	0.96	1.06	0.51	0.65	1.42
Afu6g14350	C6 transcription factor, putative	-0.18	0.35	-0.12	0.14	-0.01	0.29	0.78	0.86	0.80	1.13	0.96	0.98	1.27
Afu6g14460	2-haloalkanoic acid dehalogenase	0.07	0.30	-0.39	0.06	0.28	0.22	1.05	1.09	1.03	0.40	0.93	0.65	0.50
Afu7g00210	C6 transcription factor, putative	-0.17	-0.27	0.38	-0.06	-0.05	0.49	0.70	0.75	0.54	0.91	0.66	0.35	0.79
Afu7g00350	conserved hypothetical protein	0.20	-1.26	-0.49	0.43	-0.14	0.25	0.77	1.12	0.95	1.33	0.16	0.80	1.01
Afu7g01000	aldehyde dehydrogenase, putative	0.38	2.26	0.21	0.04	0.21	0.00	5.20	6.17	5.34	5.93	6.08	3.75	5.86
Afu7g01010	alcohol dehydrogenase, putative	0.31	0.95	0.11	-0.16	0.00	0.09	4.11	4.29	4.33	4.74	4.54	3.54	4.17
Afu7g01090	proline permease, putative	-0.03	0.55	0.08	-0.02	0.79	-0.02	1.81	2.91	2.38	1.87	2.30	1.76	2.55
Afu7g01340	RPEL repeat protein	0.10	0.38	-0.22	-0.06	0.17	0.43	1.03	1.10	0.96	1.48	1.29	0.67	1.29
Afu7g01690	acetamidase	0.20	0.36	-0.02	0.00	0.21	-0.19	0.90	1.43	1.22	0.95	1.01	0.91	1.32
Afu7g01890	C6 transcription factor, putative	0.18	-0.03	-0.22	NaN	0.19	-0.28	1.69	2.56	1.65	2.04	1.50	1.83	1.87
Afu7g02110	Ran-binding protein (RanBPM), putative	0.15	0.46	-0.10	0.25	0.13	0.43	0.82	0.89	0.79	1.33	1.12	0.68	1.35
Afu7g02390	hypothetical protein	-0.03	0.12	-0.19	0.04	-0.88	0.10	2.65	3.00	2.88	3.26	3.48	2.15	3.21
Afu7g03770	hypothetical protein	0.07	0.17	-0.11	0.02	0.07	0.09	0.75	1.22	0.79	0.84	1.04	0.84	1.16
Afu7g04260	peroxisome biosynthesis protein (Peroxin-10), putative	-0.26	-0.36	-0.23	0.08	-0.18	-0.21	0.73	1.11	0.97	0.57	0.76	0.66	0.60
Afu7g04290	amino acid permease (Gap1), putative	-0.22	0.14	-0.89	-0.23	0.22	0.22	1.91	2.13	2.31	1.90	2.91	1.77	2.59
Afu7g04340	C6 transcription factor, putative	0.06	0.97	-0.15	-0.03	0.23	-0.07	1.02	0.97	0.69	1.34	0.87	0.94	1.37
Afu7g04760	gamma-glutamyltranspeptidase	0.34	0.05	-0.33	0.16	-0.22	0.71	0.68	0.47	0.66	1.00	1.34	0.81	1.26
Afu7g06090	palmitoyl-CoA oxidase 1	0.15	-0.03	-0.23	0.08	-0.31	-0.20	1.25	0.90	0.96	0.88	0.77	NaN	0.75
Afu7g06100	acyl-coenzyme A oxidase I, peroxisomal, component A	0.05	0.45	-0.14	0.36	-0.45	0.04	1.92	2.00	2.06	1.82	2.26	1.03	1.61
Afu7g06390	MFS alpha-glucoside transporter, putative	0.49	-0.04	-0.32	-0.35	-0.02	-0.23	1.13	1.10	1.02	1.50	1.03	1.63	1.27

Afu7g06770	hypothetical protein	0.39	1.44	2.18	NaN	0.01	1.98	3.54	2.70	2.28	4.98	2.47	2.15	4.67
Afu7g08250	conserved hypothetical protein	-0.10	0.06	-0.11	0.17	0.06	-0.12	1.14	0.74	0.64	0.72	1.07	NaN	0.79
Afu8g01770	hypothetical protein	0.03	0.25	0.02	-0.09	-0.09	-0.28	0.74	1.36	0.76	1.16	0.99	1.07	1.06
Afu8g02100	beta-glucosidase, putative	0.13	0.32	0.06	0.09	0.12	0.02	1.51	1.97	1.59	1.26	2.56	1.66	1.57
Afu8g04070	glucosamine-6-phosphate deaminase, putative	0.34	0.43	0.45	NaN	0.35	-0.22	0.81	0.58	0.75	0.94	1.28	1.18	1.50
Afu8g04100	N-acetylglucosamine-6-phosphate deacetylase (NagA), putative	0.03	0.16	0.04	-0.21	0.04	0.14	0.65	0.39	0.99	0.71	1.24	1.14	1.55
Afu8g04110	DUF895 domain membrane protein	-0.03	0.05	-0.10	-0.03	0.22	-0.16	1.64	1.87	2.41	1.77	3.94	3.00	3.28
Afu8g04130	C6 transcription factor (Ctf1B), putative	0.45	0.22	0.12	-0.14	0.01	0.13	2.07	2.38	1.87	2.54	2.22	1.59	2.49
Afu8g04490	hypothetical protein	0.08	0.20	0.06	-0.02	0.07	0.07	1.35	1.13	1.12	1.57	1.50	0.91	1.54
Afu8g04780	peroxisomal membrane protein, putative	0.07	-0.19	0.15	0.28	-0.20	0.06	2.17	2.25	1.74	2.72	1.91	1.56	2.38
Afu8g05230	2-hydroxyphytanoyl-CoA lyase, putative	0.10	-0.12	-0.03	0.12	0.05	0.51	0.83	0.68	0.66	1.56	0.61	0.59	1.32
Afu8g05530	fumarate reductase (Osm1), putative	-0.11	0.04	-0.13	-0.03	-0.10	-0.26	1.24	1.58	1.26	1.49	1.60	1.13	1.80
Afu8g05710	MFS sugar transporter Stl1, putative	-1.17	-0.77	0.60	-0.11	-0.02	0.21	1.46	1.80	0.71	1.91	0.99	1.34	1.59
Afu8g07130	AhpC/TSA family thioredoxin peroxidase, putative	0.06	0.31	0.09	-0.01	0.21	-0.10	0.81	0.92	0.80	1.13	0.62	0.66	0.97

N: neutrophils from normal donors; C: neutrophils from CGD donors; H: hyphae; S: conidia

Numbers represent log2 of the ratio between fungal cells exposed to neutrophils and fungal cells that were not exposed to neutrophils
